# Soil Nutrient Enrichment Induces Trade‐Offs in Bacterial Life‐History Strategies Promoting Plant Productivity

**DOI:** 10.1002/advs.202510066

**Published:** 2025-09-18

**Authors:** Yuanyuan Yan, Xing Zhou, Liangliang Liu, Zucong Cai, Josep Penuelas, Xinqi Huang

**Affiliations:** ^1^ State Key Laboratory of Climate System Prediction and Risk Management Nanjing Normal University Nanjing 210023 China; ^2^ School of Geography Nanjing Normal University Nanjing 210023 China; ^3^ College of Life Science and Environmental Resources Yichun University Yichun 336000 China; ^4^ CREAF Campus Universitat Autònoma de Barcelona Cerdanyola del Vallès Barcelona Catalonia 08193 Spain; ^5^ Jiangsu Engineering Research Center for Soil Utilization & Sustainable Agriculture Nanjing 210023 China; ^6^ Jiangsu Center for Collaborative Innovation in Geographical Information Resource Development and Application Nanjing 210023 China

**Keywords:** bacterial community, life‐history strategy, nutrient enrichment, plant productivity, trade‐off

## Abstract

Despite the global prevalence of anthropogenic soil nutrient enrichment, its impacts on the trade‐offs in microbial life‐history strategies remain poorly understood, which is critical for agroecosystem productivity. Here, large‐scale observational studies are integrated with controlled experiments to systematically evaluate how soil nutrient enrichment affects bacterial functional potential and growth‐rate potential, ultimately determining microbial functions and plant productivity. These findings reveal stark contrasts between nutrient‐poor open field (OF) and nutrient‐rich greenhouse (GH) soils across multiple paired sites using 16S rRNA gene amplicon and metagenomic sequencing. OF microbial communities dominated by oligotrophs have higher taxonomic diversity, larger average genome sizes with abundant nutrient‐cycling genes, but lower 16S ribosomal RNA gene operon copy numbers and predicted maximum growth rates. Conversely, GH communities dominated by copiotrophs have higher growth‐rate potential, more plant‐beneficial bacteria, and higher diversity of functional genes (e.g., biofilm formation, secondary metabolism, and bacterial chemotaxis), but lower bacterial functional potential. Controlled pot experiments demonstrate that GH‐enriched microbial functions strongly promote plant growth, particularly under sufficient nutrients and abiotic stress. These findings reveal a nutrient‐driven trade‐off between bacterial functional potential and growth rate, with implications for optimizing nutrient management strategies in precision agriculture to enhance specific microbial functions and plant productivity.

## Graphical abstract

Soil nutrient enrichment induces the trade‐offs between bacterial functional potential and growth‐rate potential. Such trade‐offs favor specific plant‐beneficial species and genes, playing a pivotal role in determining plant productivity. These findings enhance the understanding of the microbial responses in agroecosystems to ongoing global environmental changes, offering theoretical foundations for sustainable agroecosystem management.

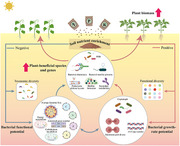

## 1 Introduction

Global fertilization with nitrogen (N), phosphorus (P), and potassium (K) has increased dramatically from 30 million tonnes in 1961 to 190 million tonnes in 2019, with N fertilization increasing the most.^[^ ^1^ ^]^ Modern agricultural systems are predominantly dependent on chemical fertilizers to sustain the escalating global demands for food.^[^ ^2^ ^]^ This reliance has led to a substantial enrichment of soil nutrients, which has become one of the most profound anthropogenic environmental disturbances to terrestrial ecosystems.^[^ ^3^ ^]^ Such nutrient enrichment can acidify soil, increase plant growth, reduce soil biota diversity, and influence the structure of microbial communities.^[^ ^2^, ^3^, ^4^ ^]^ Bacteria, as important indicators of soil health, play a crucial role in soil nutrient cycling and plant growth, and it is highly sensitive to nutrient availability.^[^ ^5^ ^]^ Recent studies are increasingly focusing on how the variability of soil nutrients affects the diversity and composition of bacterial communities.^[^ ^3^, ^6^, ^7^ ^]^ For example, N and P enrichment can compromise multiple soil ecosystem functions and weaken the relationship between soil biodiversity and ecosystem functions.^[^ ^3^ ^]^ To achieve sustainable agricultural development, the mechanisms of complex soil nutrient‐microbe interactions in agroecosystems need to be fully understood.

Critical knowledge gaps remain regarding how changes in soil nutrients affect microbial life‐history strategies, which are essential for guiding agroecosystem production. Growth rate, a fundamental property of microbes, is the basis for many ecosystem functions.^[^ ^8^ ^]^ The predicted growth‐rate potential is the main feature for distinguishing rhizosphere and soil bacteria, and it is also the most important genomic predictor that determines the ability of bacteria to colonize the rhizosphere.^[^ ^9^ ^]^ The number of ribosomal RNA gene operons (*rrn*) in bacterial genomes represents a phylogenetically conserved genomic trait at the genus and species levels strongly associated with bacterial growth rate and nutrient‐acquisition strategies.^[^ ^10^ ^]^ A recent study highlighted the importance of soil properties, particularly soil pH, soil organic carbon, and extracellular enzymes, in driving microbial growth strategies during grassland restoration.^[^ ^11^ ^]^ Previous studies have also identified trade‐offs between microbial growth rate and genome size.^[^ ^12^, ^13^, ^14^ ^]^ Genome size reflects both genetic and functional diversity due to its strong positive correlation with gene number.^[^ ^15^, ^16^, ^17^ ^]^ For instance, Wang et al.^[^ ^17^ ^]^ found that bacterial average genome size was strongly negatively correlated with soil pH in the forest ecosystems. Similarly, Bahram et al.^[^ ^18^ ^]^ revealed that soil pH was the essential driver of bacterial average genome size in the global topsoil. Although these findings collectively establish how microbial life‐history strategies respond to various environmental factors, the role of soil nutrients in mediating these strategies remains to be systematically investigated.

The transition of microbial life history strategies is inevitably accompanied by shifts in functional traits.^[^ ^19^ ^]^ Soil microorganisms mediate essential biogeochemical cycles, including the decomposition of organic carbon (C), N fixation, nitrification/denitrification, P solubilization, and sulfur (S) transformation.^[^ ^20^, ^21^, ^22^ ^]^ Notably, variation in soil nutrient concentrations (particularly N, P, and K) can cause microbe‐mediated alterations in the cycling of elements. For example, long‐term inputs of N suppress phosphatase‐mediated P mineralization, and long‐term inputs of P increase microbial P fixation by enhancing polyphosphate synthesis.^[^ ^23^, ^24^, ^25^, ^26^ ^]^ A recent study also demonstrated that adding N and P significantly reduced ecosystem multifunctionality, particularly functions involved in ecosystem stability and the cycling of C and N.^[^ ^3^ ^]^ These functional shifts affect C sequestration, the availability of plant nutrients, and overall ecosystem productivity.^[^ ^27^ ^]^ In addition to nutrient cycling, soil microorganisms play a crucial role in promoting plant growth by protecting plants from both abiotic and biotic stresses through direct and indirect mechanisms.^[^ ^28^, ^29^ ^]^ Direct mechanisms include the formation of biofilms, which facilitate microbial nutrient acquisition, and indirect mechanisms involve the biological control of plant pathogens by the synthesis of secondary metabolites such as antibiotics.^[^ ^30^, ^31^ ^]^ Limited research, however, has explored how soil nutrients (N, P, and K) mediate the balance between the microbial cycling of elements and functions promoting plant growth. This knowledge gap limits our ability to develop sustainable agricultural practices that optimize both crop yields and soil ecosystem health.

Here, we first conducted a large‐scale comparative analysis of bacterial communities in agricultural soils under contrasting nutrient conditions by collecting paired open field (OF, nutrient‐poor) and greenhouse (GH, nutrient‐rich) soils. We integrated 16S rRNA gene sequencing with metagenomic analysis to systematically evaluate bacterial growth‐rate potential (e.g., *rrn* copy number, maximum growth rate) and functional potential (e.g., average genome size, nutrient‐cycling genes, plant‐beneficial genes). We subsequently conducted controlled experiments to investigate how these changes in microbial communities influenced plant productivity to verify the outcomes of the shifts in microbial functions under nutrient enrichment. Plants were cultivated in soils inoculated with microbial communities originating from either nutrient‐rich or nutrient‐poor environments. Specifically, our research framework systematically addressed three principal objectives: i) to identify shifts in bacterial life‐history strategies driven by soil nutrients, ii) to assess the functional traits of bacterial communities in response to changes in soil nutrients, and iii) to determine the effects of these changes on plant performance. We hypothesized that: i) nutrient‐rich soils would harbor copiotrophic bacterial communities with higher growth rates compared to nutrient‐poor soils; ii) nutrient‐rich soils would exhibit reduced bacterial functional potential; iii) the shift in microbial community composition and functions would positively influence plant productivity under nutrient‐rich conditions and abiotic stress. These findings would advance our understanding of the connections between soil nutrients, microbes, and plants, providing insights for sustainable agricultural practices that enhance the health of soil ecosystems.

## 2 Results

### 2.1 Difference in Bacterial Communities Between OF and GH Soils

**Figure 1:**
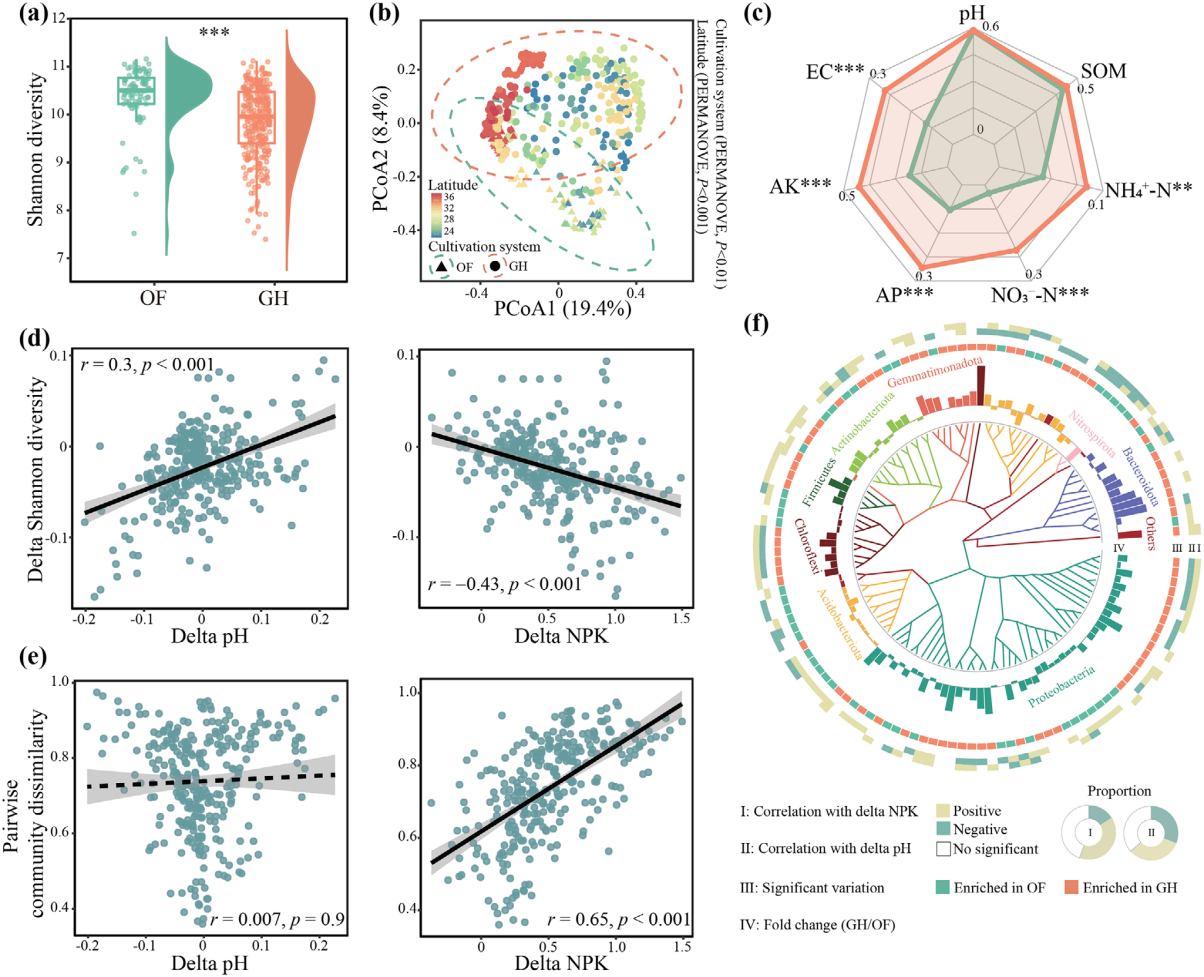
Soil bacterial communities based on 16S rRNA amplicon data in the open field (OF) and greenhouse (GH) soils. a) Difference in bacterial Shannon diversity between the OF and GH soils (Wilcoxon rank‐sum test; ***, *p* < 0.001). b) Principal coordinates analysis (PCoA) based on Bray‐Curtis distances showing differences in bacterial community structure between OF and GH soils. *p*‐values were determined using PERMANOVA. c) Radar plots of the differences in properties between the OF and GH soils. pH, soil pH; EC, soil electrical conductivity; SOM, soil organic matter; AP, available phosphorus; AK, available potassium; NH_4_^+^–N, ammonium nitrogen; NO_3_^−^–N, nitrate nitrogen. Asterisks denote significant differences between the OF and GH soils (Wilcoxon rank‐sum test; ** and *** represent *p* < 0.01 and < 0.001, respectively). d) Relationships between delta Shannon diversity and delta soil pH and NPK concentration. Delta is calculated from the logarithm‐transformed (log_10_) ratio of pairwise GH to OF soils from the same sites. e) Relationships between community dissimilarity of pairwise GH and OF soils and delta soil pH and NPK concentration. The black lines represent ordinary least squares linear regressions. The gray areas represent the 95% confidence intervals. f) Phylogenetic tree of bacterial zOTUs significantly (adjusted *p* < 0.05) enriched in the OF or GH soils based on the ANCOM‐BC2 analysis. All *p*‐values were adjusted using the Benjamini‐Hochberg false discovery rate procedure. The zOTUs with average relative abundances >0.05% in the OF and GH soils were retained in the figure. Taxonomic information, zOTU enrichment, the difference in the relative abundances of zOTUs between the OF and GH soils, the relationships between the delta relative abundances of the zOTUs, delta pH, and NPK concentration are displayed from the inner to the outer circles. Pie plots show the ratios of zOTUs correlated with delta pH and NPK concentration.

Bacterial alpha diversity, quantified using the Shannon index, was significantly (Wilcoxon test, *p* < 0.001) lower by 4.96% in the GH compared to the OF soils (**Figure** **1**a). A principal coordinate analysis (PCoA) indicated that the bacterial‐community structure of the OF and GH soils formed distinct clusters in ordination spaces (Figure 1b, Permutational multivariate analysis of variance, PERMANOVA *p* < 0.01). Latitude was also a significant determinant (PERMANOVA, *p* < 0.001) of bacterial‐community variation. The abundances of phyla Bacteroidota, Firmicutes, Proteobacteria, and Gemmatimonadota were generally higher in the GH soils, whereas abundances of Chloroflexi, Acidobacteria, and Nitrospirales were generally higher in the OF soils (Figure S2, Supporting Information). NPK concentration in the GH soils was significantly (Wilcoxon test, *p* < 0.001) higher by an average of 221.1% compared to the OF soils (Figure S3, Supporting Information), and the ammonium nitrogen (NH_4_^+^–N), nitrate nitrogen (NO_3_^−^–N), available phosphorus (AP), and available potassium (AK) concentrations were significantly (Wilcoxon test, *p* < 0.01) higher by 88.4, 681.6, 211.6, and 125.7%, respectively (Figure 1c). Soil electric conductivity (EC) in the GH soils was significantly (Wilcoxon test, *p* < 0.001) higher by 139.3% compared to the OF soils (Figure 1c), soil pH and the concentration of soil organic matter (SOM), however, did not differ significantly (Wilcoxon test, *p* > 0.05) between the two soils (Figure 1c). Linear least‐squares regression modeling identified contrasting biogeochemical drivers: the variation in Shannon diversity was correlated positively with the difference in soil pH (*p* < 0.001) but negatively with the difference in NPK concentration (*p* < 0.001) between the GH and OF soils (Figure 1d). Similar results were further observed in OF pairs or GH pairs (Figure S4a,c,e,g, Supporting Information). A pairwise analysis of community dissimilarity demonstrated that the NPK concentration (*p* < 0.001) had a more important role than soil pH (*p* > 0.05) in affecting the bacterial communities (Figure 1e). For OF or GH pairs: increased soil pH and nutrient variability corresponded to greater bacterial community dissimilarity (Figure S4b,d,f,h, Supporting Information). The variation in the relative abundances of the dominant bacterial taxa was primarily associated with the difference in soil pH and NPK concentration between the GH and OF soils, with notably greater positive relationships with the difference in NPK concentration (Figure 1f; Figure S5, Supporting Information).

The community similarities were significantly (*p* < 0.001) negatively correlated with both geographic distance and the edaphic distance within the OF and GH soils, but the slopes of distance‐decay relationships (DDRs) were notably steeper (*p* < 0.001) for the OF than the GH soils (Figure S6a, Supporting Information), indicating that the GH soils had a weaker distance‐decay relationship and a lower beta diversity. A null model (Figure S6b, Supporting Information) and a neutral community model (NCM) (Figure S6c, Supporting Information) were used to assess the contributions of deterministic and stochastic processes in bacterial‐community assembly. Stochastic processes (|β‐nearest taxon index, βNTI| < 2) were the dominant assembly processes structuring the bacterial communities in both the OF and GH soils, with a relative importance of 62.31% and 66.79% in the OF and GH soils, respectively (Figure S6b, Supporting Information). The NCM analysis indicated that the explained variation was greater for the GH (*R* ^2^ = 0.52) than the OF (*R* ^2^ = 0.41) soils (Figure S6c, Supporting Information). These results from the null model and NCM indicated that bacterial‐community assembly was primarily governed by stochastic processes, particularly for the GH soils.

### 2.2 Difference in Bacterial Functional Potential Between the OF and GH Soils

**Figure 2:**
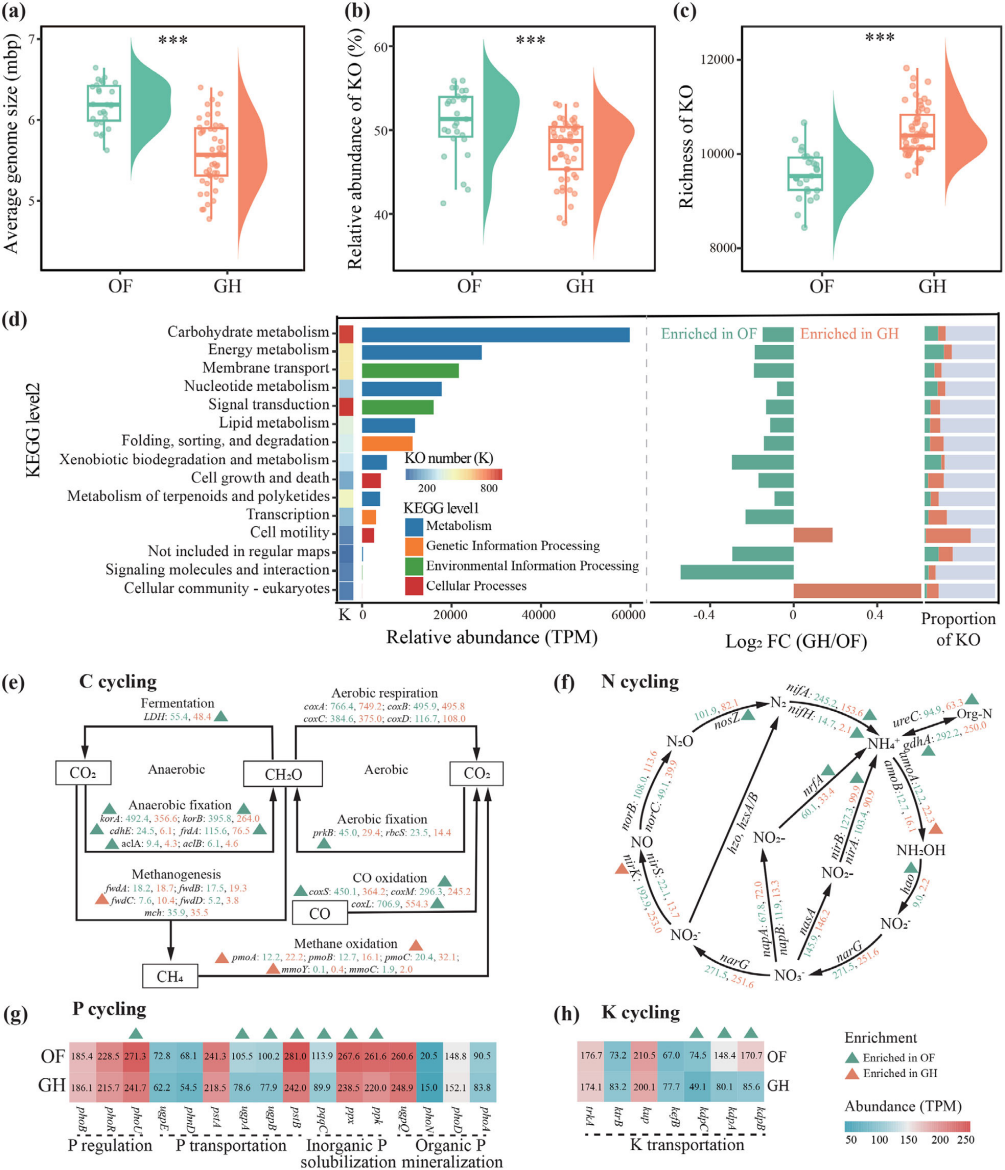
Bacterial functional potential based on metagenomic data in the open field (OF) and greenhouse (GH) soils. a–c) Raincloud plots showing the differences in average size of bacterial genomes (a), relative abundance of reads mapping to Kyoto Encyclopedia of Genes and Genomes (KEGG) Ontology (KOs) (b), and richness of KOs (c) between the OF and GH soils. In the box plot, the center line represents the median, box edges show the 25th and 75th percentiles, and whiskers extend to 1.5× the interquartile range. Asterisks indicate significant differences between the OF and GH soils (Wilcoxon rank‐sum test; ***, *p* < 0.001). d) The differences in the abundance of KEGG level2 pathways between the OF and GH soils. Only those KEGG pathways that were significantly more abundant in the OF soils (green) or GH soils (orange) in the Wilcoxon rank‐sum test (FDR‐adjusted *p* < 0.05) are shown. The stacked bar chart shows the proportions of KOs significantly enriched in the GH and OF soils. e–h) Differences between the OF and GH soils in the abundances of functional genes involved in the cycling of C (e), N (f), P (g), and K (h). Green triangles indicate significantly enriched genes in the OF soils, and orange triangles indicate significantly enriched genes in the GH soils (Wilcoxon sum rank test, FDR‐adjusted *p* < 0.05).

The average bacterial genome size was 9.67% smaller (Wilcoxon test, *p* < 0.001) in the GH than the OF soils (**Figure** **2**a). The PCoA analysis identified a significant (PERMANOVA, *p* < 0.001) difference in functional‐gene composition between the OF and GH soils (Figure S7a, Supporting Information). The total relative abundances of reads mapping to genes annotated in the Kyoto Encyclopedia of Genes and Genomes (KEGG) Ontology (KO) ranged from 38.90 to 55.89% among all OF and GH soils and were significantly (Wilcoxon test, *p* < 0.001) lower by 6.46% in the GH soils compared to the OF soils (Figure 2b). In contrast, the richness of the KO genes was significantly (Wilcoxon test, *p* < 0.001) higher by 9.57% in the GH soils compared to the OF soils (Figure 2c). A volcano plot indicated that the relative abundances of 1695 KO genes were significantly (|log2 fold change| > 1 and FDR‐adjusted *p* < 0.05) higher in the GH soils, and the abundances of 589 KO genes were notably (|log2 fold change| > 1 and FDR‐adjusted *p* < 0.05) higher in the OF soils (Figure S7b, Supporting Information). The OF soils notably maintained a significantly (Wilcoxon test, FDR‐adjusted *p* < 0.05) higher metabolic potential compared to the GH soils, including the metabolism of carbohydrates, energy, nucleotides, and lipids (Figure 2d). The number of KO genes in most of the KEGG pathways, however, was significantly higher in the GH than the OF soils. An analysis of specific genes for cycling C, N, P, K, and S indicated that the abundances of most of these genes were higher in the OF than the GH soils (Figure 2e–h; Figure S8, Supporting Information). Anaerobic fixation, aerobic fixation, and CO oxidation processes for cycling C were lower for the GH soils, indicated by significantly (Wilcoxon test, FDR adjusted *p* < 0.05) lower abundances of the *korA*, *korB*, *cdhE*, *frdA*, *aclA*, *prkB*, *coxS*, *coxM*, and *coxL* genes, but the abundances of genes involved in methane oxidation (*pmoA*, *pmoC*, and *mmoY*) were significantly (Wilcoxon test, FDR adjusted *p* < 0.05) higher in the GH than the OF soils (Figure 2e). The abundances of genes involved in N fixation (*nifA* and *nifH*), nitrite reduction (*nrfA* and *nirB*), and mineralization (*gdhA* and *ureC*) were significantly (Wilcoxon test, FDR adjusted *p* < 0.05) higher in the OF soils, but the abundances of genes involved in nitrification (*amoA* were significantly (Wilcoxon test, FDR adjusted *p* < 0.05) higher in the GH soils (Figure 2f). Genes associated with P regulation (*phoU*), P transportation (*ugpA*, *ugpB*, and *pstB*), and inorganic P solubilization (*pqqC*, *ppk*, and *ppX*) were more (Wilcoxon test, FDR adjusted *p* < 0.05) abundant in the OF soils (Figure 2g). The abundances of marker genes *kdpA*, *kdpB*, and *kdpC* involved in K transportation were significantly (Wilcoxon test, FDR‐adjusted *p* < 0.05) higher in the OF than the GH soils (Figure 2h). The analysis of S cycling also indicated that the abundances of genes involved in dissimilatory sulfate reduction (*aprA*, *aprB*, *dsrA*, and *dsrB*) were significantly (Wilcoxon test, FDR‐adjusted *p* < 0.05) lower in the GH than the OF soils (Figure S8, Supporting Information).

We observed that microbial functional potential was negatively correlated with NPK concentration, whereas bacterial growth‐rate potential showed positive associations (Figure S9, Supporting Information). Linear least‐squares regression indicated that the difference in NPK concentration determined the variation in overall bacterial functional potential between the GH and OF soils (Figure S10, Supporting Information). Specifically, the variation in average bacterial genome size and the abundance of KO genes were significantly negatively correlated with the difference in NPK concentration, and the variation in the richness of the KO genes was significantly positively correlated with the difference in NPK concentration. A correlation heatmap indicated that the variation in the abundances of most genes associated with the cycling of C, N, P, K, and S was correlated negatively with the differences in the concentrations of NH_4_^+^–N, NO_3_^−^–N, AP, and AK but positively with the difference in soil pH (Figure S11, Supporting Information).

### 2.3 Difference in Bacterial Growth‐Rate Potential and Plant‐Beneficial Species and Genes Between the OF and GH Soils

**Figure 3:**
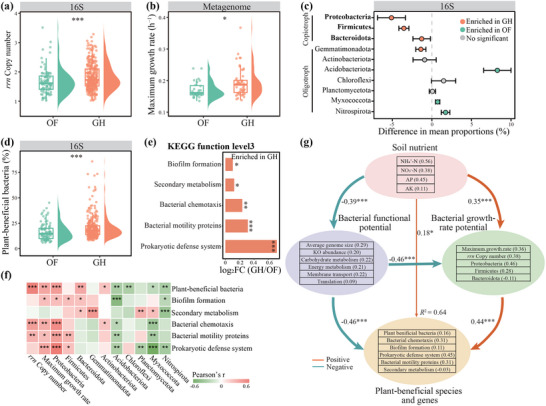
Bacterial growth‐rate potential and plant‐beneficial species and genes in the open field (OF) and greenhouse (GH) soils. a) Differences in bacterial *rrn* copy number based on 16S rRNA amplicon data between the OF and GH soils (Wilcoxon rank‐sum test; ***, *p* < 0.001). b) Differences in estimated maximum bacterial growth rate between the OF and GH soils (Wilcoxon rank‐sum test; *, *p* < 0.05). Maximum bacterial growth rates were detected by analyzing the metagenomic data. c) Differences in the relative abundances of bacterial phyla between the OF and GH soils. Bacterial phyla are detected by 16S rRNA gene amplicon sequencing. Copiotrophs are labeled in bold. d) Differences in the relative abundances of plant‐beneficial bacteria based on 16S rRNA amplicon data between the OF and GH soils (Wilcoxon rank‐sum test; ***, *p* < 0.001). e) Beneficial Kyoto Encyclopedia of Genes and Genomes pathways significantly enriched in the GH soils compared to the OF soils (Wilcoxon rank‐sum test; *, *p* < 0.05; **, *p* < 0.01; ***, *p* < 0.001). f) Pearson's correlation coefficients between bacterial growth‐rate potential indicators (*rrn* copy number and maximum growth rate estimated from metagenomics contigs), the relative abundances of bacterial phyla, and the relative abundances of specific plant‐beneficial species and genes. *, *p* < 0.05; **, *p* < 0.01; ***, *p* < 0.001. g) Direct and indirect effects of factors driving plant‐beneficial species and genes using Partial Least Squares Structural Equation Modeling (*n* = 78). The ellipses represent the latent variables, and the rectangles represent the observed variables. The values in parentheses indicate the weight of the indicator. The red and green arrows in the PLS‐SEM indicate positive and negative relationships, respectively. *, *p* < 0.05; ***, *p* < 0.001.

We estimated *rrn* copy number using data from both amplicon and metagenomic sequencing. The results indicated that *rrn* copy numbers of both 16S rRNA gene amplicon (average increase of 9.32%) and metagenomic data (average increase of 4.28%) were significantly (Wilcoxon test, *p* < 0.05) higher in the GH than the OF soils (**Figure** **3**a; Figure S12a, Supporting Information). Similarly, the estimated bacterial maximum growth rate was notably (Wilcoxon test, *p* < 0.05, average increase of 12.55%) higher in the GH than the OF soils (Figure 3b). The maximum growth rate was significantly positively correlated with *rrn* copy number (Figure S12b, Supporting Information), whereas average genome size was significantly negatively correlated with both *rrn* copy number and maximum growth rate (Figure S12c,d, Supporting Information). The variations in *rrn* copy number and maximum growth rate were positively correlated with the difference in soil NPK concentrations between the GH and OF soils (Figure S13, Supporting Information), indicating that nutrient enrichment may accelerate bacterial growth. Similar results were observed in the paired OF and the paired GH soils (Figure S14, Supporting Information). The relative abundances of phyla, primarily classified as copiotrophs such as Proteobacteria, Firmicutes, and Bacteroidota, were notably (Wilcoxon test, *p* < 0.05) higher in the GH soils (Figure 3c). Additionally, the estimated average *rrn* copy number of these copiotrophic bacteria (Bacteroidota: 3.79, Firmicutes: 9.66, Proteobacteria: 2.49) exceeded those of most oligotrophic bacteria (1.18–2.60, Figure S15, Supporting Information).

The relative abundances of plant‐beneficial bacteria (PBB), including biocontrol agents, bacteria promoting plant growth, and stress‐resistant bacteria, were significantly (Wilcoxon test, *p* < 0.01, average increase of 32.73%, 17.99%, and 88.67%) higher in the GH than the OF soils (Figure 3d; Figure S16a, Supporting Information). The variation in the relative abundance of PBB was significantly and positively correlated to the difference in soil NPK concentration between the GH and OF soils (Figure S16b, Supporting Information). Significantly positive relationships between variation in PBB relative abundance and soil NPK concentration differences were also observed in both OF and GH soils (Figure S17, Supporting Information). These PBB taxa primarily belonged to copiotrophic phyla such as Proteobacteria, Firmicutes, and Bacteroidota, collectively accounting for 68.59% of the total numbers (Figure S18, Supporting Information). These plant‐beneficial genes were notably significantly more common in the GH than the OF soils, including biofilm formation, secondary metabolism, bacterial chemotaxis, bacterial motility proteins, and prokaryotic defensive systems (Figure 3e). These specific plant‐beneficial genes were positively correlated with both the relative abundance of copiotrophs and bacterial growth‐rate potential (Figure 3f).

Partial least squares structural equation modeling (PLS‐SEM) identified the direct and indirect effects of the NPK nutrients, bacterial functional potential, and bacterial growth‐rate potential on specific plant‐beneficial species and genes (Figure 3g). The PLS‐SEM accounted for 64% of the variance in these specific plant‐beneficial species and genes. The soil nutrients notably had a direct positive effect on specific plant‐beneficial species and genes. Higher nutrient concentrations induced a trade‐off between bacterial functional potential and growth‐rate potential, with positive effects on specific plant‐beneficial species and genes. The soil nutrient concentrations were correlated positively with bacterial growth rate but negatively with the bacterial functional potential. The bacterial growth rate was also positively correlated with specific species and genes, but the bacterial functional potential was negatively correlated with specific plant‐beneficial species and genes.

### 2.4 Properties of Metagenome Assembled Genomes (MAGs) Between the OF and GH Soils

**Figure 4:**
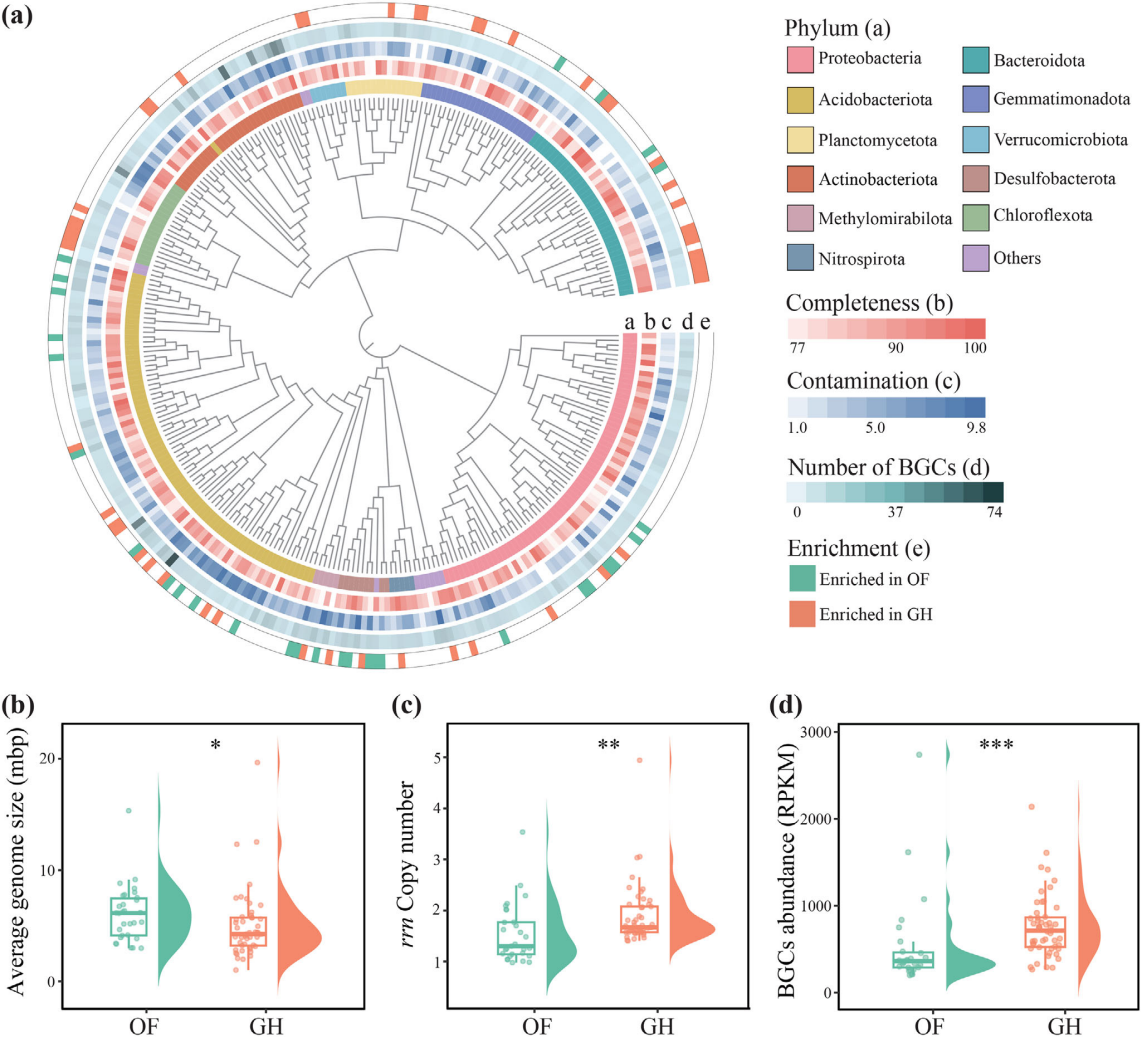
Summary of the metagenome‐assembled genomes (MAGs) in the open field (OF) and greenhouse (GH) soils. a) Phylogenetic tree of MAGs, taxonomic information, MAG completeness, MAG contamination, the number of biosynthetic gene clusters (BGCs) defined by antiSMASH, MAG abundances in the OF and GH soils, and MAG enrichment are displayed from the inner to the outer circles. b–d) Differences in average genome size, *rrn* copy number, and BGCs abundance in the MAGs enriched in OF or GH soils. Asterisks denote significant differences between the OF and GH soils (Wilcoxon rank‐sum test; *, *p* < 0.05; **, *p* < 0.01; ***, *p* < 0.001).

A total of 301 high‐quality bacterial MAGs (>70% completeness and <10% contamination) were reconstructed from the metagenomic data (**Figure** **4**a). These MAGs primarily belonged to the phyla Proteobacteria, Acidobacteriota, Bacteroidota, Actinobacteriota, Gemmatimonadota, Chloroflexota, and Planctomycetota. The average genome size of the MAGs was significantly (Wilcoxon test, *p* < 0.05) smaller in the GH than the OF soils (Figure 4b), whereas the *rrn* copy number was significantly (Wilcoxon test, *p* < 0.001) higher (Figure 4c). Secondary metabolism, biofilm formation, bacterial chemotaxis, bacterial motility proteins, and prokaryotic defensive systems of the MAGs were more common in the GH than the OF soils (Figure S19, Supporting Information). These 301 bacterial MAGs collectively encoded 1964 biosynthetic gene clusters (BGCs), with a higher abundance in the GH than the OF soils (Figure 4d). The BGCs involved in plant growth and health, such as nonribosomal peptide synthetase (NRPS), type I polyketide synthase (T1RKS), and type III polyketide synthase (T3RKS), were significantly more abundant in the GH than the OF soils (Figure S20, Supporting Information).

### 2.5 Verification using Controlled Pot Experiments

**Figure 5:**
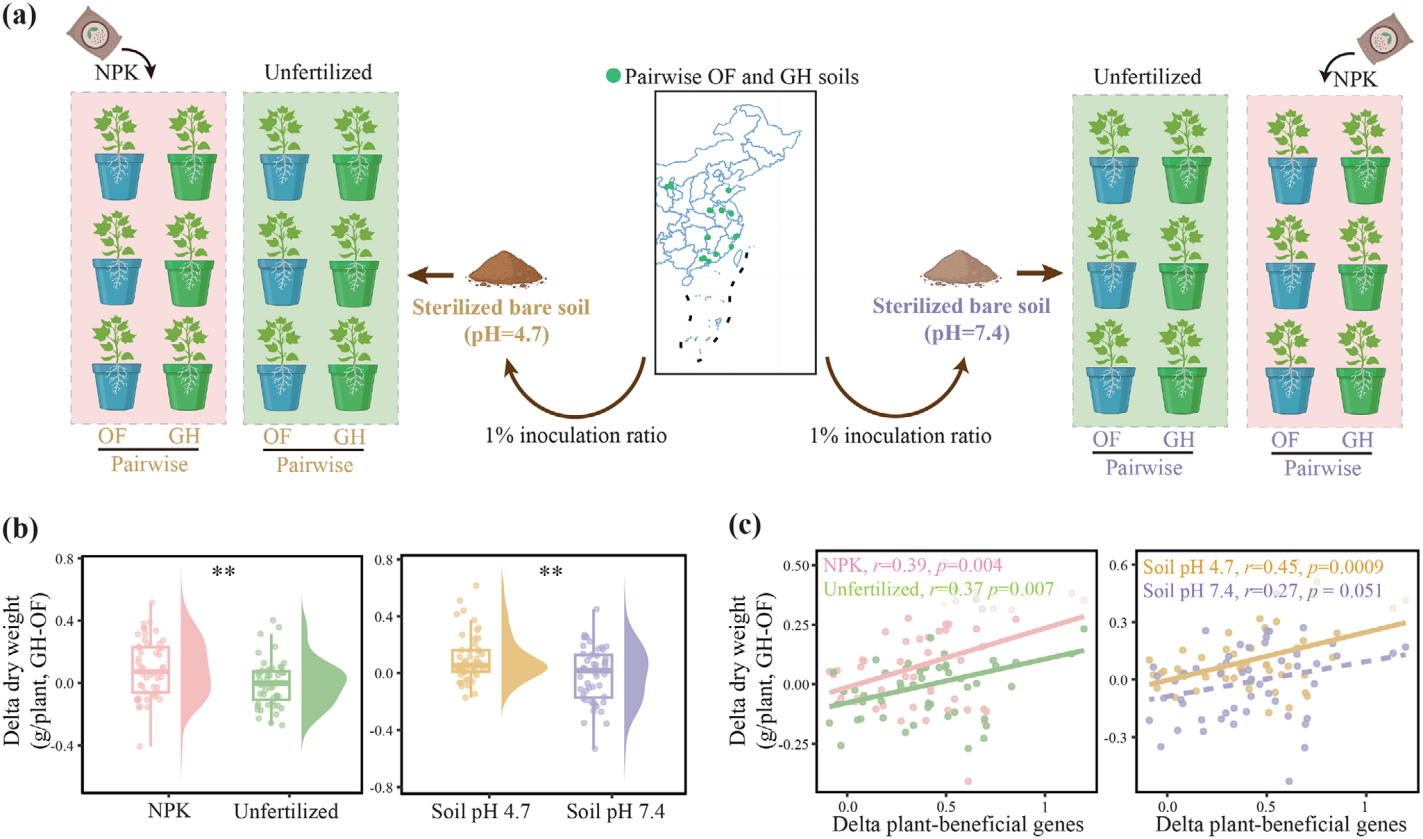
Effects of microbial consortium in the open field (OF) and greenhouse (GH) soils on plant productivity. a) Design of the controlled pot experiment. b) Delta (GH‐OF) dry weight of plants of inoculated pairwise GH and OF consortia in the NPK‐fertilized (NPK) and unfertilized treatments under different soil pH conditions (Wilcoxon rank‐sum test). c) Spearman's correlations between the delta dry weight of plants and the delta plant‐beneficial genes between inoculated pairwise GH and OF consortium. Specific plant‐beneficial genes involve those properties listed in Figure 3. Delta‐specific plant‐beneficial genes are calculated from the logarithm‐transformed (log_10_) ratio of pairwise GH to OF soils.

We conducted pot experiments under different nutrient conditions (fertilized vs unfertilized treatments) and environmental stresses (acidic soil vs neutral soil) to investigate the effects of the bacterial communities and functions on plant growth (**Figure** **5**a). The difference in dry weight of plant between the GH and the OF soils was significantly (Wilcoxon test, *p* < 0.01) higher in the NPK fertilized treatment than in the unfertilized treatment, suggesting that the microorganisms had greater plant‐promoting effects under a nutrient‐rich environment in the GH than the OF soils (Figure 5b). The difference in dry weight of the plant was notably (Wilcoxon test, *p* < 0.01) higher in an acidic environment (soil pH = 4.7) than in a neutral environment (soil pH = 7.4), indicating that the plant‐promoting effect of the microorganisms was greater in the GH than the OF soils under abiotic stress. The differences in dry weight of the plant were positively correlated with the variation in specific plant‐beneficial genes, with stronger correlations in the NPK fertilized treatment and the acidic environment (Figure 5c).

## 3 Discussion

### 3.1 Nutrient Enrichment Decreased Soil Bacterial α and β Diversities

Identifying the interplay of soil factors and microbial associations across spatiotemporal gradients remains a fundamental challenge in advancing soil ecology.^[^ ^32^ ^]^ The impact of nutrient enrichment on soil microbial communities has garnered increasing attention in recent years. Previous studies have demonstrated that N enrichment in natural grassland ecosystems tends to reduce the diversity of soil bacteria and nematodes.^[^ ^33^, ^34^ ^]^ Similarly, a 13‐year field experiment in an agricultural ecosystem revealed that N enrichment significantly reduced the diversity of bacteria, fungi, and nematodes.^[^ ^3^ ^]^ Unlike prior studies that primarily focused on specific sites or regions, our large‐scale investigation demonstrated that soil nutrient (N, P, and K) enrichment, from traditional agriculture to intensive greenhouse cultivation, significantly reduced bacterial alpha diversity across diverse climatic and soil conditions. Several factors may contribute to this observed reduction in microbial diversity. A previous study demonstrated that the decline in bacterial diversity was strongly linked to the reduction in soil pH caused by elevated N inputs.^[^ ^34^ ^]^ However, our findings revealed that no significant differences in soil pH between the nutrient‐rich GH soils and the nutrient‐poor OF soils. Instead, nutrient enrichment may alter microbial interactions, shifting them from mutualism to competition and favoring the dominance of efficient and competitive microbial species, ultimately decreasing community diversity.^[^ ^3^, ^35^ ^]^ The loss of microbial diversity can impair multiple ecosystem functions, including nutrient cycling, decomposition, plant production, and pathogen control.^[^ ^36^ ^]^ Our results demonstrated that nutrient enrichment in agricultural ecosystems pervasively reduced the alpha diversity of bacterial communities at large spatial scales.

Soil microbial communities are generally characterized by strong spatial heterogeneity, which ensures microbial beta diversity.^[^ ^37^, ^38^ ^]^ In the present study, we observed that latitude was a key driver of bacterial beta diversity. This latitudinal distribution pattern of beta diversity is primarily governed by climatic variables and soil heterogeneity.^[^ ^18^, ^39^, ^40^, ^41^ ^]^ A Previous study revealed that temperature was more important than soil heterogeneity in mediating microbial beta diversity.^[^ ^39^ ^]^ Previous studies, however, have provided extensive evidence that land‐use conversion has led to soil biotic homogenization.^[^ ^42^, ^43^, ^44^ ^]^ A recent study combining continental survey results with a global meta‐analysis demonstrated that the conversion of natural ecosystems (forest, grassland, and wetland) to agricultural land led to the homogenization of soil bacterial taxa.^[^ ^43^ ^]^ Another study found that the transition from grassland to arable land led to fungal homogenization, with prevalent fungal species becoming more abundant and rare groups becoming less abundant or absent in arable land.^[^ ^42^ ^]^ Our study revealed a weaker distance‐decay pattern of bacterial‐community similarity in the nutrient‐rich GH soils compared to the nutrient‐poor OF soils, suggesting that nutrient enrichment drives the homogenization of bacterial communities in agricultural soil. Nutrient enrichment can decrease soil heterogeneity, leading to the disappearance of oligotrophic species and the dominance of copiotrophic species, which may be key drivers of bacterial‐community homogenization.^[^ ^42^, ^45^ ^]^ Collectively, these results indicated that high‐intensity anthropogenic interference can induce biotic homogenization and biodiversity loss in terrestrial ecosystems.

### 3.2 Nutrient Enrichment Decreased Soil Bacterial Functional Potential

Functional traits better reflect microbial responses to resource shifts and stress than taxonomy.^[^ ^17^, ^46^, ^47^ ^]^ Our findings indicated that nutrient enrichment significantly decreased the average bacterial genome size, a trait closely associated with microbial lifestyle and functional potential.^[^ ^48^, ^49^ ^]^ Previous studies have suggested that slow‐growing oligotrophic bacteria may have large genomes, enabling them to thrive in resource‐limited environments.^[^ ^50^, ^51^ ^]^ For instance, a grassland study demonstrated that N and P additions led to a notable decrease in the average genome size of the bacterial communities, with copiotrophic bacteria exhibiting smaller genomes.^[^ ^5^ ^]^ These results align with our observation that the average bacterial genome size was significantly and negatively correlated with the concentrations of soil nutrients. However, research in an arid ecosystem found no significant differences in the average size of bacterial genomes between bare soils with poor resources and vegetated soils with rich resources.^[^ ^52^ ^]^ This discrepancy suggests an interactive effect of abiotic stress and nutrient enrichment on bacterial genome size. Notably, nutrient enrichment increased functional diversity while decreasing taxonomic diversity, suggesting the decoupling of these two facets of diversity, consistent with previous findings along a wide pH gradient.^[^ ^17^ ^]^ In nutrient‐poor environments, oligotrophic bacteria prioritize nutrient acquisition (e.g., enriched membrane transport pathways, Figure 2d), enrich specific metabolic genes, potentially leading to low functional diversity.^[^ ^53^, ^54^ ^]^ In contrast, copiotrophic microbes in nutrient‐rich environments display more diverse genes involved in cell division, the cell cycle, and RNA metabolism, in addition to their basic metabolism.^[^ ^53^ ^]^ Similarly, a global study demonstrated the decoupling of functional and phylogenetic diversity in plant communities across diverse climatic conditions,^[^ ^55^ ^]^ highlighting the widespread prevalence of phylogenetic–functional decoupling across the biosphere under environmental changes.

Our findings indicated that nutrient enrichment significantly reduced the relative abundances of functional genes for C, N, P, K, and S cycling. Previous studies have shown that the land‐use conversion from steppe to cropland increased functional genes for labile C, but decreased those involved in the mobilization of recalcitrant substrates (e.g. lignin), as well as P and S cycling.^[^ ^47^ ^]^ Similarly, a global‐scale study demonstrated that the agricultural conversion of natural ecosystems diminished N fixation, P mineralization, and transport potential.^[^ ^43^ ^]^ Despite established impacts of land‐use on nutrient‐cycling functions, the underlying drivers remain poorly understood. We found negative correlations between the relative abundances of element‐cycling genes and soil NH_4_^+^–N, NO_3_^−^–N, and AP concentrations, which may be attributed to excessive fertilization reducing the reliance of microbes on genes associated with nutrient acquisition.^[^ ^43^ ^]^ For example, in our study, these genes related to N fixation (*nifH* and *nifA*) and the solubilization of inorganic P (*pqqC*, *ppx*, *ppk*) were significantly more abundant in the nutrient‐poor OF soils than the nutrient‐rich GH soils. This pattern was likely due to the application of inorganic N and P fertilizers, consistent with previous studies.^[^ ^31^, ^56^, ^57^ ^]^ Conversely, the input of ammonium‐N fertilizer stimulated nitrification,^[^ ^58^ ^]^ as evidenced by the higher abundances of the *amoA* and *amoB* genes in the nutrient‐rich GH soils. This aligns with a previous study showing that nutrient‐deficient conditions enhance the compensatory colonization of nutrient cycling‐related endophytes in the rhizosphere.^[^ ^59^ ^]^ Collectively, these results suggest that soil nutrient enrichment in intensive agricultural systems substantially impairs bacterial functional potential, particularly functions involved in nutrient cycling.

### 3.3 Nutrient Enrichment Increased Soil Bacterial Growth‐Rate Potential

Microbial life‐history strategies play a pivotal role in determining their metabolic potential and response to environmental changes.^[^ ^19^ ^]^ The copiotroph–oligotroph dichotomy is widely used for classifying microbial life‐history strategies.^[^ ^52^ ^]^ Copiotrophs grow faster and rely on high resource availability, whereas oligotrophs use resources more efficiently at the expense of a low growth rate.^[^ ^54^ ^]^ In our study, bacterial communities in the nutrient‐rich GH soils displayed higher *rrn* copy numbers and were enriched in copiotrophic bacteria, such as Firmicutes, Proteobacteria, and Bacteroidota, compared to those in the nutrient‐poor OF soils. A similar global study suggested that the community‐level *rrn* copy numbers were significantly higher in coastal sediments than in ocean water, and were positively correlated with the concentrations of N and P.^[^ ^45^ ^]^ We also found that the predicted maximum growth rate of microbial communities was notably higher in the nutrient‐rich soils than in the nutrient‐poor soils, consistent with these findings. This observation aligned with a previous study indicating that microbial communities in vegetated soils with abundant resources exhibited higher predicted maximum growth rates than those in bare soils with fewer resources.^[^ ^52^ ^]^ Microbial species with higher growth‐rate potentials are generally more competitive within their ecological niches. For instance, rhizospheric bacteria have higher growth‐rate potentials than do bulk soil bacteria, enabling them to successfully colonize the rhizosphere.^[^ ^9^ ^]^ In conclusion, our results demonstrate that nutrient enrichment significantly enhances microbial growth‐rate potential, offering critical insights into how practices of agricultural management can influence soil microbial life‐history strategies.

Together, we observed that the trade‐offs between bacterial functional potential and growth‐rate potential are induced by soil nutrient enrichment. Similarly, a recent study revealed that microbes use nutrients as signals to assess their environments and implement trade‐offs between rapid growth and preparation for changing environments.^[^ ^60^ ^]^ For example, bacteria can accelerate cell growth via upregulating ribosome synthesis and downregulating non‐required metabolic proteins following an increase in nutrients.^[^ ^61^ ^]^ This mechanistic understanding aligns with observed ecological patterns: microbes in nutrient‐limited primary forests predominantly employ resource‐acquisition strategies, whereas those in resource‐rich secondary forests exhibit high growth traits.^[^ ^62^ ^]^ These findings collectively support the notion that nutrient fluctuations drive trade‐offs between bacterial growth and adaptability, and between growth and survival.^[^ ^60^, ^63^ ^]^ In other words, improvements in adaptability and survival often come at the expense of reduced growth rates, resulting in trade‐offs. Under other selective pressures, a recent study suggested that increased pesticide diversity drove bacteria to streamline their average genome size to conserve energy while enhancing C, N, P, and S metabolic capacities, leading to the trade‐offs.^[^ ^64^ ^]^ This finding contrasts with our observation that average genome size varies inversely with element cycling functions, underscoring the complexity of trade‐offs in microbial life history strategies when confronted with diverse environmental changes.

### 3.4 Nutrient Enrichment Enriched Specific Plant‐Beneficial Species and Genes to Promote Plant Productivity

In addition to its impacts on bacterial‐community composition, functional potential, and growth rate, our study demonstrated that nutrient enrichment also enhanced specific plant‐beneficial species and genes. Using the plant‐beneficial bacteria (PBB) database, which curates experimentally validated beneficial bacteria,^[^ ^65^ ^]^ we found that the nutrient‐rich GH soils were enriched in PBB. Notably, most of these enriched PBB were copiotrophs, consistent with recent findings,^[^ ^65^ ^]^ suggesting connections between microbial growth rates and plant‐beneficial species. Our metagenomic analysis further revealed that nutrient enrichment increased specific plant‐beneficial genes, including biofilm formation, bacterial chemotaxis, secondary metabolism such as the biosynthesis of nonribosomal peptides and polyketides. The formation of biofilms serves as a crucial adaptive strategy for microorganisms, functioning as an effective biological tool to alleviate plant stress by enhancing multi‐stress resilience.^[^ ^30^ ^]^ Chemotaxis enables bacteria to detect and transport nutrients efficiently, thereby promoting efficient nutrient uptake, distribution, and ultimately plant growth.^[^ ^66^ ^]^ Additionally, bacterial chemotaxis plays a crucial role in biofilm formation by facilitating bacterial interactions with the environment and other species.^[^ ^67^ ^]^ Secondary metabolism enhances plant nutrient uptake, growth, and stress resistance.^[^ ^31^, ^68^ ^]^ To validate these findings, we conducted verification experiments, which confirmed that the enriched plant‐beneficial bacteria and genes significantly contributed to increased plant biomass, particularly under nutrient‐rich environments and acidic stress. Collectively, our results provide experimental evidence that soil nutrient enrichment enhances specific plant‐beneficial species and genes, thereby protecting plants from abiotic stress and improving plant productivity.

## 4 Conclusion

**Figure 6:**
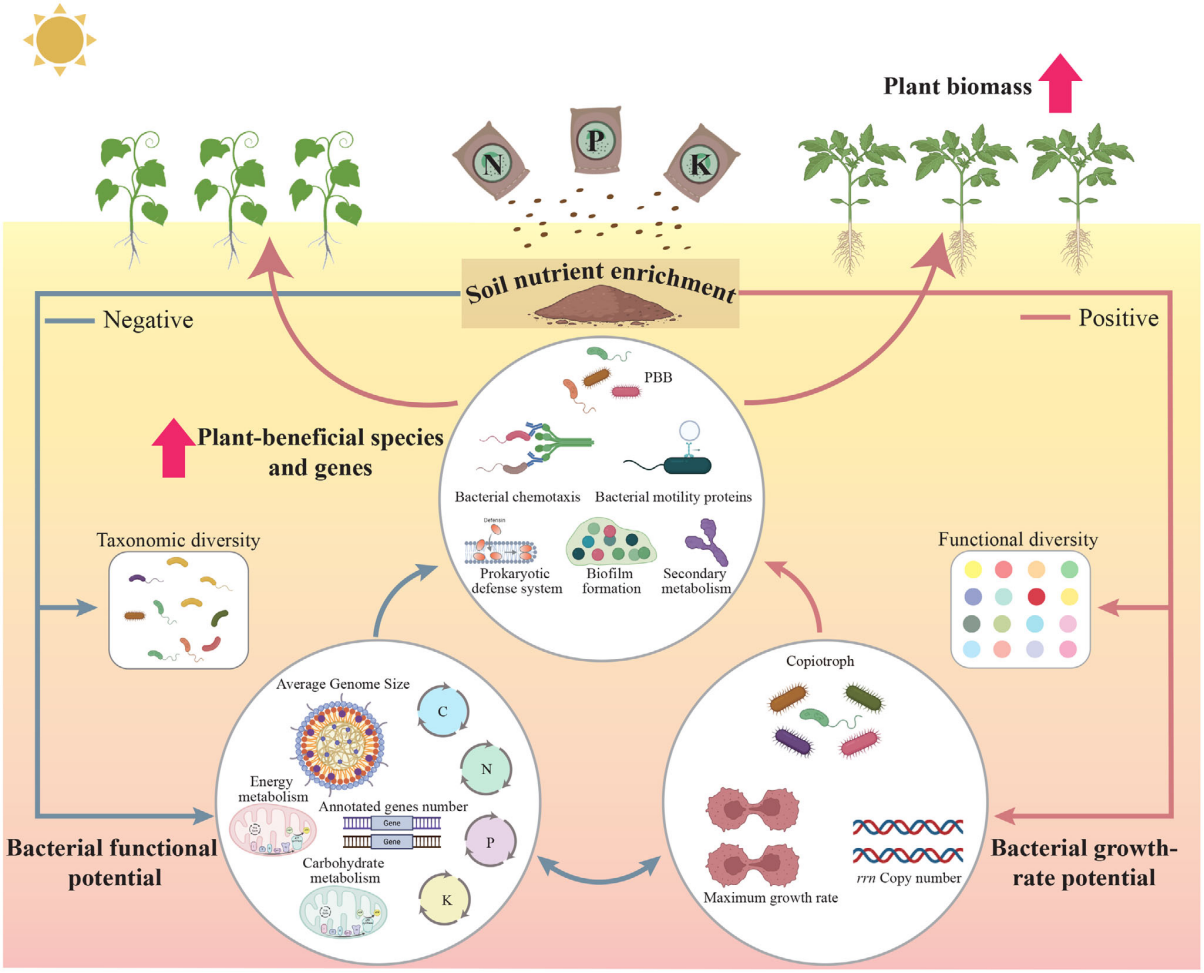
Conceptual diagram of the variations in bacterial functional potential, growth‐rate potential, and plant‐beneficial species and genes in response to soil nutrient enrichment and their impacts on plant productivity. C, carbon‐cycling genes; N, nitrogen‐cycling genes; P, phosphorus‐cycling genes; K, potassium‐cycling genes. PBB, plant‐beneficial bacteria.

This study provides the first evidence that trade‐offs between bacterial functional potential and growth‐rate potential, driven by soil nutrient enrichment, play a pivotal role in determining plant productivity (**Figure** **6**). Oligotrophic bacteria, which exhibit high diversity, were enriched in the OF soils with limited NPK nutrients. These oligotrophs possessed larger genomes and harbored more nutrient‐cycling functional genes, enabling them to adopt resource‐acquisition strategies at the expense of reduced growth rates. In contrast, nutrient‐rich GH soils exhibited lower biodiversity but harbored a greater abundance of copiotrophic bacteria. These copiotrophs were characterized by higher growth rates and a more diverse set of functional genes, including those associated with biofilm formation, secondary metabolism, bacterial chemotaxis, and bacterial motility proteins. These enhanced functions facilitated interactions with both bacteria and plants, thereby supporting plant growth. However, as a trade‐off, these copiotrophs downregulated functional potential that was non‐essential for nutrient acquisition in the nutrient‐rich soils. Our findings generally underscore the critical importance of trade‐offs in bacterial life‐history strategies induced by nutrient enrichment in determining specific plant‐beneficial species and genes. These results suggest that adequate soil nutrients are conducive to regulating the functions of soil microorganisms, enabling them to shift from elemental cycling to promoting plant growth. Future research should focus on elucidating the interactions between bacteria and eukaryotic microorganisms, such as fungi and predatory protists, in nutrient‐gradient environments. These interactions may be pivotal in shaping the soil's overall functions as well as specific microbial processes that support plant growth.

## 5 Experimental Section

### Soil Sampling

Soil samples were controlled from 89 agricultural sites in nine provinces based on their representativeness in agricultural production, covering a distance of 1878 km across China (Figure S1, Supporting Information). These sites were climatically diverse, with mean annual temperature ranging from 8.2 to 22.3 °C and mean annual precipitation ranging from 194.6 to 1852.5 mm. Each site comprised paired samples of OF and GH cultivation systems separated by less than 100 m. The OF systems are characterized by low land‐use intensity and minimal chemical inputs, representing traditional agricultural systems with poor nutrients. In contrast, the GH systems are developed from OF systems, are marked by high land‐use intensity and substantial chemical inputs, representing modern and intensive agricultural systems with rich nutrients. The average input of chemical fertilizer in GH systems exceeds fourfold that of OF systems.^[^ ^69^ ^]^ GH soils are highly heterogeneous, so three to four samples of GH soil were collected to match the OF soil at each site. Topsoil samples (0–20 cm layer) for each OF and GH soil were collected from nine random locations following standardized protocols, yielding a total of 396 samples of ≈300 g each (89 OF and 307 GH samples). The fresh soil collected was sieved through a 2‐mm mesh to remove plant roots and debris and was fully mixed to ensure homogeneity. Each sample was subsequently divided into two portions: one portion was stored at −80 °C until DNA extraction, and the other portion was stored at −4 °C for physicochemical analysis.

### Analysis of Soil Variables

Soil pH and EC were measured using S220k and S230 meters (Mettler, Switzerland) at soil‐to‐water ratios of 1:2.5 and 1:5 (w/v), respectively. The concentration of SOM was determined using wet digestion with H_2_SO_4_‐K_2_Cr_2_O_7_.^[^ ^70^ ^]^ NH_4_^+^–N and NO_3_^−^–N were extracted using a 1 mol L^−1^ KCl solution at a 1:5 (w/v) ratio, with samples shaken at 250 r min^−1^ for 1 h before filtering for 30 min, and the concentration of the filtrate was analyzed using a continuous flow analyzer (San++; Skalar, Netherlands). Soil AP was extracted using a 0.5 mol L^−1^ NaHCO_3_ solution at a 1:20 (w/v) ratio, and the concentration of the filtrate was determined by the molybdenum–antimony colorimetric method. Soil AK was extracted using a 1 mol L^−1^ CH_3_COONH_4_ solution at a 1:10 (w/v) ratio, and the concentration of the filtrate was analyzed using flame photometry.

### DNA Extraction, Amplicon Sequencing, and Data Processing

DNA was extracted from 0.5 g of each soil sample using the MP FastDNA SPIN Kit (MP Biomedicals, Santa Ana, CA, USA) following the manufacturer's instructions. The extracted DNA was dissolved in 100 µL of elution buffer, and its concentration and purity were determined using a DS‐11 spectrophotometer (Denovix Inc., Wilmington, DE, USA). The bacterial 16S rRNA gene was amplified by PCR using the forward primer 515F (GTGCCAGCMGCCGCGG) and the reverse primer 907R (CCGTCAATTCMTTTRAGTTT) attached to adapter sequences and barcode sequences. The purified amplicons were sequenced on an Illumina MiSeq platform (Majorbio Bio‐Pharm Technology Co. Ltd., Shanghai, China).

The raw sequences were filtered for quality using USEARCH.^[^ ^71^ ^]^ The primer sequences and low‐quality reads with quality scores <30 were first excised, followed by the consolidation of paired‐end sequences. The resultant sequences were quality‐filtered (with a maximum expected error rate of 0.5), and singletons were eliminated using USEARCH. The UNOISE3^[^ ^72^ ^]^ algorithm, used with default parameters within USEARCH, was used to generate zero‐radius operational taxonomic units (zOTUs). The number of reads retained in these samples ranged from 55,347 to 267,859 (Table S1, Supporting Information). Representative bacterial zOTU sequences were classified using the SILVA database (http://www.arb‐silva.de), release 138. Nonbacterial zOTUs (chloroplast, mitochondrial, and viridiplantae) were then removed. The total number of bacterial zOTUs across all samples was 38,101.

Based on taxonomy information derived from the 16S rRNA amplicon data, the Plant‐Beneficial Bacteria (PBB) database was utilized to identify plant‐beneficial bacterial taxa.^[^ ^65^ ^]^ Like other taxonomy‐based functional annotation databases, the PBB database primarily operates at the genus level. The number of *rrn* copies was estimated in the bacterial zOTUs using the *rrn*DB database (https://rrndb.umms.med.umich.edu). Each zOTU was matched with the database, starting from the lowest taxonomic rank. The mean *rrn* copy number was used for all corresponding child taxa for the zOTUs with available matches. If no matches were available, matches with higher ranks were sought, and the mean *rrn* copy number of the parent taxa was assigned to the zOTU. To determine the community‐level *rrn* copy number for each sample, the mean of the estimated *rrn* copy numbers weighted by the relative abundance of each zOTU was calculated.

### Metagenome Sequencing and Data Processing

The metagenomes of the soil samples were sequenced on an Illumina HiSeq 2500 platform (Magigene Co. Ltd, Shenzhen, China). To improve the reliability of the raw data, the quality of the raw sequencing data was evaluated using FastQC v0.11.9 (https://github.com/s‐andrews/FastQC), and low‐quality reads were filtered out using Trimmomatic v0.39^[^ ^73^ ^]^ (LEADING:3, TRAILING:3, SLIDINGWINDOW:5:20, MINLEN:50), for a total of 2184 GB of clean reads. The number of reads retained per sample after quality trimming ranged from 32 369 097 to 68 948 343 (Table S1, Supporting Information). These clean reads from all samples were co‐assembled into contigs using Megahit v1.2.9,^[^ ^74^ ^]^ and prodigal v2.6.3^[^ ^75^ ^]^ was used to predict open reading frames from assembled contigs longer than 500 bp. The assembly generated a total of 45 265 020 contigs, with a combined length of 44 567 607 622 bp. Redundant genes were removed, and a non‐redundant gene catalog from all samples was obtained using MMseqs2 v15.6f452^[^ ^76^ ^]^ with the parameter ‐e 0.001 –min‐seq‐id 0.95 ‐c 0.90. Gene abundance (transcripts per million, TPM) was calculated by mapping clean reads to the nonredundant gene catalog using Salmon v1.8.0.^[^ ^77^ ^]^ Protein‐coding sequences were then annotated against the eggNOG v5.0.2 database (http://eggnog5.embl.de) for functional annotation using Diamond v2.1.9.^[^ ^78^ ^]^ For KEGG‐annotated metagenomic results, quantified the richness of KOs was quantified by counting the number of unique KOs with at least one mapped read per sample. Beneficial KEGG functions based on established literature were also identified.^[^ ^30^, ^31^, ^66^, ^67^, ^68^ ^]^

From the metagenomic contigs, a set of traits including average 16S rRNA copy number was calculated, average genome size, and maximum growth rate (Figures 2 and 3). The average 16S rRNA copy number and average genome size were calculated as described by Pereira‐Flores et al.^[^ ^16^ ^]^ Specifically, the average 16S rRNA copy number was derived by dividing the coverage of the 16S rRNA gene by the number of genomes in a metagenome. The 16S rRNA genes were annotated using SortMeRNA 2.0,^[^ ^79^ ^]^ and their coverage was defined as the number of annotated base pairs divided by the length of 16S rRNA gene. The number of genomes was estimated based on the mean coverage of the 35 single‐copy genes, which were identified and summarized in previous studies.^[^ ^16^, ^80^ ^]^ The average genome size was estimated as the number of base pairs divided by the number of genomes in a metagenome. The minimum generation time was calculated using the “metagenome_V2” (“MMv2”) version of gRodon models, as described by Weissman et al.^[^ ^81^ ^]^ Briefly, this method quantifies the codon usage bias in highly expressed ribosomal protein genes while accounting for the relative coverages of genes in the community. The method has previously been shown to be the most accurate when genes are predicted from assembled contigs.^[^ ^82^ ^]^ Ribosomal protein genes were identified from the predicted coding regions in contigs using blastn v2.9.0,^[^ ^83^ ^]^ with the reference database being the previously constructed ribosomal protein gene sequence database.^[^ ^84^ ^]^ The relative coverages of genes were determined by aligning sequence reads with predicted coding regions using bwa mem v0.7.17^[^ ^85^ ^]^ and quantifying gene coverages using samtools v1.8.0.^[^ ^77^ ^]^ Finally, the maximum growth rate (h^−1^) was determined as the inverse of the minimum generation time.

For all samples, assembled contigs were subsequently binned into metagenome‐assembled genomes (MAGs) using metaBAT2 v2.12.1.^[^ ^86^ ^]^ The completeness and contamination of the MAGs were evaluated using CheckM v1.2.3,^[^ ^87^ ^]^ and low‐quality MAGs (completeness <70% and contamination >10%) were filtered out according to previous studies.^[^ ^88^, ^89^ ^]^ Retained high‐quality MAGs were dereplicated using dRep v3.5.0.^[^ ^90^ ^]^ With the parameter ‐sa 0.95, generating 301 unique MAGs. The abundances of the MAGs were quantified using Coverm v0.7.0 (https://github.com/wwood/CoverM), and taxonomic information was annotated, and a phylogenetic tree was constructed using GTDB‐Tk v2.3.2.^[^ ^91^ ^]^ with the Genome Taxonomy Database Release 207. The phylogenetic tree was visualized using iTOL (https://itol.embl.de/). The process of functional annotation for the MAGs was consistent with that used for the contigs. Biosynthetic gene clusters (BGCs) are important types of gene sets commonly found in the genomes of diverse organisms, where they play crucial metabolic and regulatory roles.^[^ ^92^ ^]^ These BGCs primarily direct the synthesis of secondary metabolites, such as antibiotics, antifungals, and siderophores that mediate communication, competition, and interactions with other organisms and the environment.^[^ ^93^, ^94^ ^]^ A previous study demonstrated that such secondary metabolites can enhance plant resistance to abiotic stress.^[^ ^95^ ^]^ Therefore, identifying BGCs and predicting their encoded products and functions is essential for understanding plant–microbe interactions. The BGCs in the high‐quality MAGs were identified using antiSMASH v.7.1.0.^[^ ^96^ ^]^ The processes and parameters used to calculate the *rrn* copy number and average genome size of MAGs were consistent with those applied to contigs.

### Microcosmic Experiment

Pot experiments were conducted to investigate the effects of the soil bacterial communities and functions on plant growth under different nutrient and pH conditions (Figure 5a). A total of 78 OF and GH soils from the nine provinces were selected, with three OF soils and six paired GH soils from each province (only three GH soils in one of the provinces). Two types of bare soils were collected from Jiangxi and Jiangsu provinces for microbial inoculation, with pH values of 4.7 and 7.4, to create abiotic stress and non‐stress environments, respectively. Both soils had minimal nutrient concentrations: NH_4_^+^–N, 12.6 and 6.05 mg kg^−1^; NO_3_^−^–N, 17.7 and 11.8 mg kg^−1^; AP, 12.3 and 21.2 mg kg^−1^; and AK, 60.5 and 73.7 mg kg^−1^. These two soils were sterilized by 50 kGy γ‐irradiation. One gram of OF or GH soil was inoculated into 100 g of sterilized bare soil mixed with sterilized vermiculite (2:1, v/v), according to previous studies.^[^ ^97^, ^98^ ^]^ Following a 3‐week incubation, a cucumber (Jinyou 409) or tomato (Provence) seedling was transplanted into each pot for a 4‐week cultivation under NPK‐fertilized and unfertilized conditions. The fertilization treatment consisted of five applications of chemical fertilizer, with a total application of 100 mg kg^−1^ of N, P_2_O_5_, and K_2_O. The NPK concentration was inherently higher for the GH than the OF soils, but the 1% inoculation ratio eliminated nutrient differences between the soils after inoculation, because it was negligible compared to the fertilization nutrients, allowing us to investigate the effects of the soil microbial communities and functions on plant growth, eliminating the interference from nutrient differences. All treatments were cultivated in a climate‐controlled environment (16 h during the day at 30 °C and 8 h during the night at 25 °C). The dry weight of 1872 whole plants (78 soil types × 2 soil pH levels × 2 plant species × 2 fertilization treatments × 3 replicates) was ultimately measured.

### Statistical Analyses

All statistical analyses were performed using R v4.3.1 (https://www.r‐project.org/). The alpha diversity of the bacterial communities was estimated using the Shannon index. A principal coordinate analysis based on Bray–Curtis distances was performed to visualize the differences in bacterial beta diversity. A permutational multivariate analysis of variance was performed to examine the effects of cultivation system and latitude on bacterial beta diversity. Relationships between pairwise bacterial alpha and beta diversities and delta soil properties were assessed using linear least‐squares regression with the “ggpubr” R package. Delta was calculated from the logarithm‐transformed (log_10_) ratio of GH to OF: delta = log_10_ (X_GH_/X_OF_), where X_GH_ and X_OF_ are variables of the pairwise GH and OF soils, respectively. Specifically, delta NPK concentration is the average of the natural‐logarithm‐transformed ratios of the concentrations of NH_4_^+^–N, NO_3_^−^–N, AP, and AK. The phylogenetic tree of bacterial zOTUs was visualized using the R packages of “ggtree” and “ggtreeExtra.” To investigate the differences in bacterial‐community compositions, the Wilcoxon rank‐sum test from the “stats” R package was used to identify significant differences in the relative abundance of each zOTU (>0.01%) between the OF and GH soils, and the results were visualized using TreeMap. All *p*‐values for multiple testing were corrected using the Benjamini–Hochberg method.^[^ ^99^ ^]^

Distance‐decay relationships (DDRs) were calculated to evaluate the distributional patterns of the bacterial communities between the geographic and edaphic distance and community similarities (1 – Bray–Curtis distance). The geographic distances among the sampling sites were calculated based on the sampling coordinates (latitude and longitude). The edaphic distances among samples were calculated using soil physiochemical properties based on Euclidean distance. The difference in the slopes of the DDRs was calculated using the “simba” R package. The β‐nearest taxon index and the Raup–Crick index were used to determine the relative importance of the deterministic and stochastic processes governing ecological bacterial‐community assembly, as described in a previous study.^[^ ^100^ ^]^ A neutral community model was used to determine the potential importance of the stochastic processes to bacterial‐community assembly.^[^ ^101^ ^]^

To analyze the differences in bacterial functional potential and growth‐rate potential between the OF and GH soils, the differences between the OF and GH soils were evaluated in average genome size, *rrn* copy number, maximum growth rate, total abundances of categories of functional genes, richness of categories of functional genes, the relative abundance of specific plant‐beneficial genes, and the relative abundance of functional genes involved in the cycling of C, N, P, K, and S using the Wilcoxon rank‐sum test. The widely applied r‐ and K‐selection categories of soil microbial communities were used to identify the differences in copiotrophs and oligotrophs between the OF and GH soils. A heatmap of Pearson correlation coefficients between specific plant‐beneficial species and genes, bacterial taxa, and growth‐rate potential was constructed using the “pheatmap” R package. Partial Least Squares Structural equation modeling (PLS‐SEM) was employed to explore the driving factors directly and indirectly affecting plant‐beneficial species and genes. PLS‐SEM is particularly suitable for complex models with numerous latent variables, as it relaxes distributional assumptions regarding samples and error terms.^[^ ^102^ ^]^ The PLS‐SEM incorporated four latent variables, including soil nutrients (NH_4_^+^–N, NO_3_^−^–N, AP, and AK), bacterial functional potential (average genome size, total KO abundance, relative abundances of KEGG pathways), bacterial growth‐rate potential (maximum growth rate and *rrn* copy number derived from metagenomic data, the relative abundances of copiotrophic phyla), and plant‐beneficial species and genes (relative abundances of PBB, biofilm formation, secondary metabolism, bacterial chemotaxis, bacterial motility proteins, and prokaryotic defensive systems). These variables were selected based on their strong correlations. The PLS‐SEM was performed using the “plspm” R package. Linear least‐squares regressions were performed to analyze the relationships between delta bacterial functional potential, growth‐rate potential, and delta NPK concentration.

### Ethics Approval and Consent to Participate

This study did not involve human or animal subjects, and thus, no ethical approval was required. The study protocol adhered to the guidelines established by the journal.

## Conflict of Interest

The authors declare no conflict of interest.

## Author Contributions

Z.C. and X.H. designed research; Y.Y., X.Z., and L.L. performed research. Y.Y. and X.Z. contributed to new reagents/analytic tools. Y.Y. and X.Z. wrote the paper. J.P., Z.C., and X.H. revised the paper.

## Supporting information



Supporting Information

## Data Availability

The 16S rRNA gene sequences and the metagenomic sequences were submitted to the NCBI Sequence Read Archive (SRA) database under the accession numbers PRJNA1214025 and PRJNA1161440, respectively. The data supporting the findings of this study are available within the paper and its Supplementary file.
