# Supplementary material for: Soil Nutrient Enrichment Induces Trade‐Offs in Bacterial Life‐History Strategies Promoting Plant Productivity
Source: Adv Sci (Weinh). 2025 Sep 18;12(45):e10066. doi: 10.1002/advs.202510066 (PMC12677649; doi:10.1002/advs.202510066)
Supplement: Supplementary file 1 — Supporting Information [file ADVS-12-e10066-s001.docx]

Supporting Information

**Soil Nutrient Enrichment Induces Trade-Offs in Bacterial Life-History Strategies Promoting Plant Productivity**

*Yuanyuan Yan, Xing Zhou, Liangliang Liu, Zucong Cai, Josep Penuelas, Xinqi Huang*


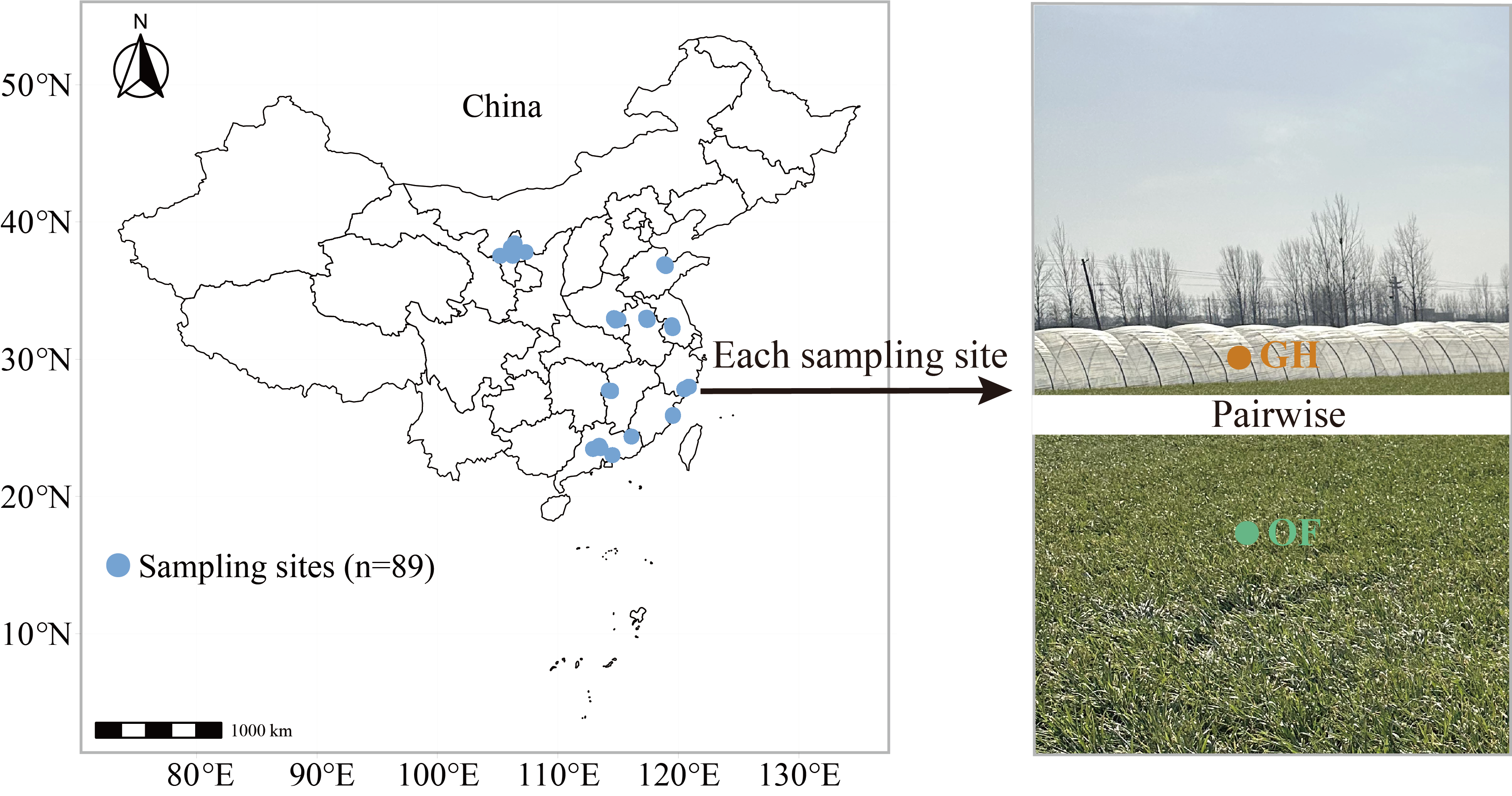


**Figure S1**. Locations of the 89 sampling sites. Pairwise OF and GH soils were collected from each site. Considering the high heterogeneity of GH soils, three to four samples of GH soil were collected for matching the OF soil at each site, resulting in 89 OF and 307 GH soils in total.


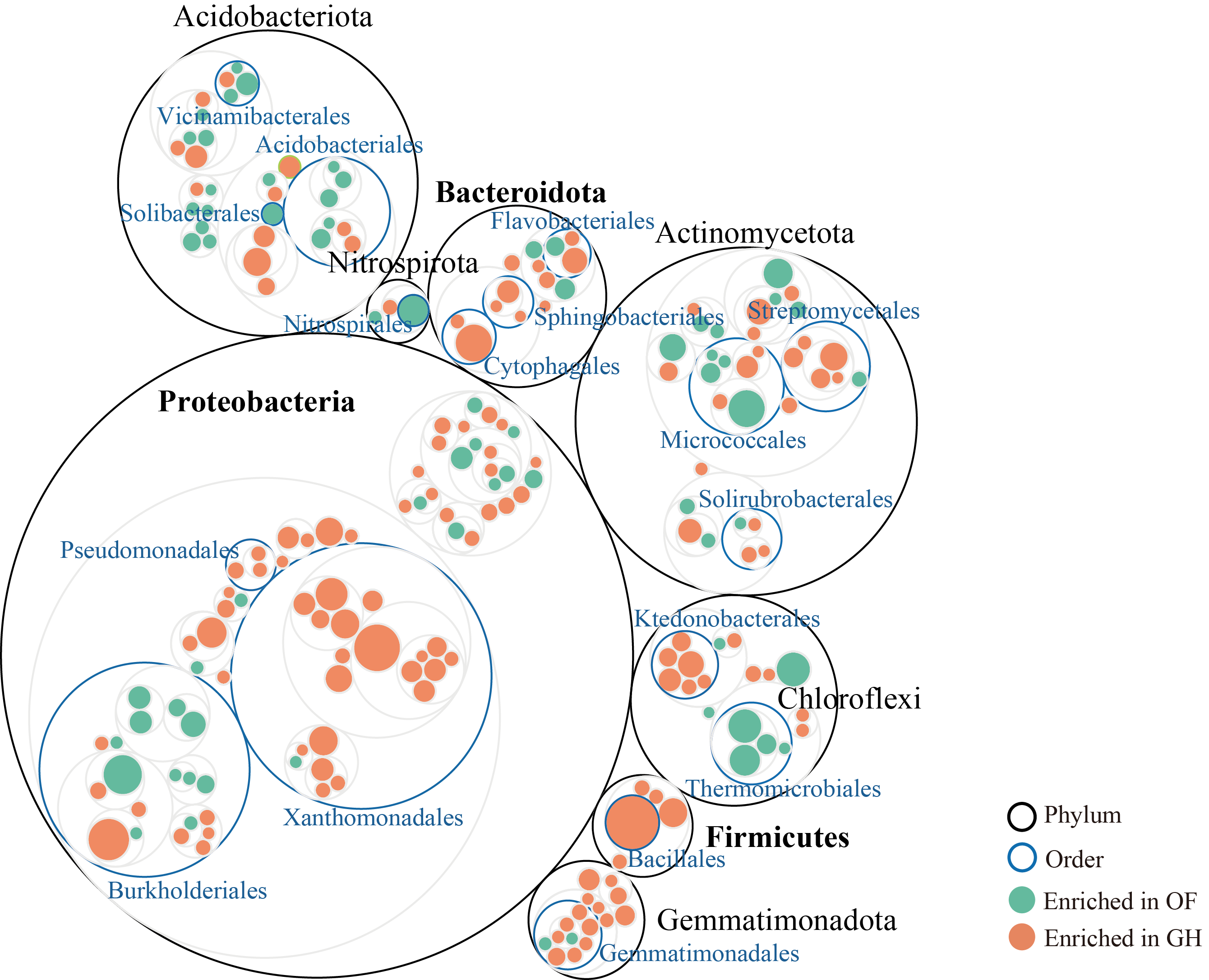


**Figure S2**. Differences in the relative abundances of bacterial taxa between the open field (OF) and greenhouse (GH) soils (*n* = 396). Green and orange bubbles represent zOTUs significantly enriched (Wilcoxon rank-sum test, FDR adjusted *p* < 0.05) in the OF and GH soils, respectively. Bubble size represents the relative abundance of the zOTUs in the OF and GH soils.

**
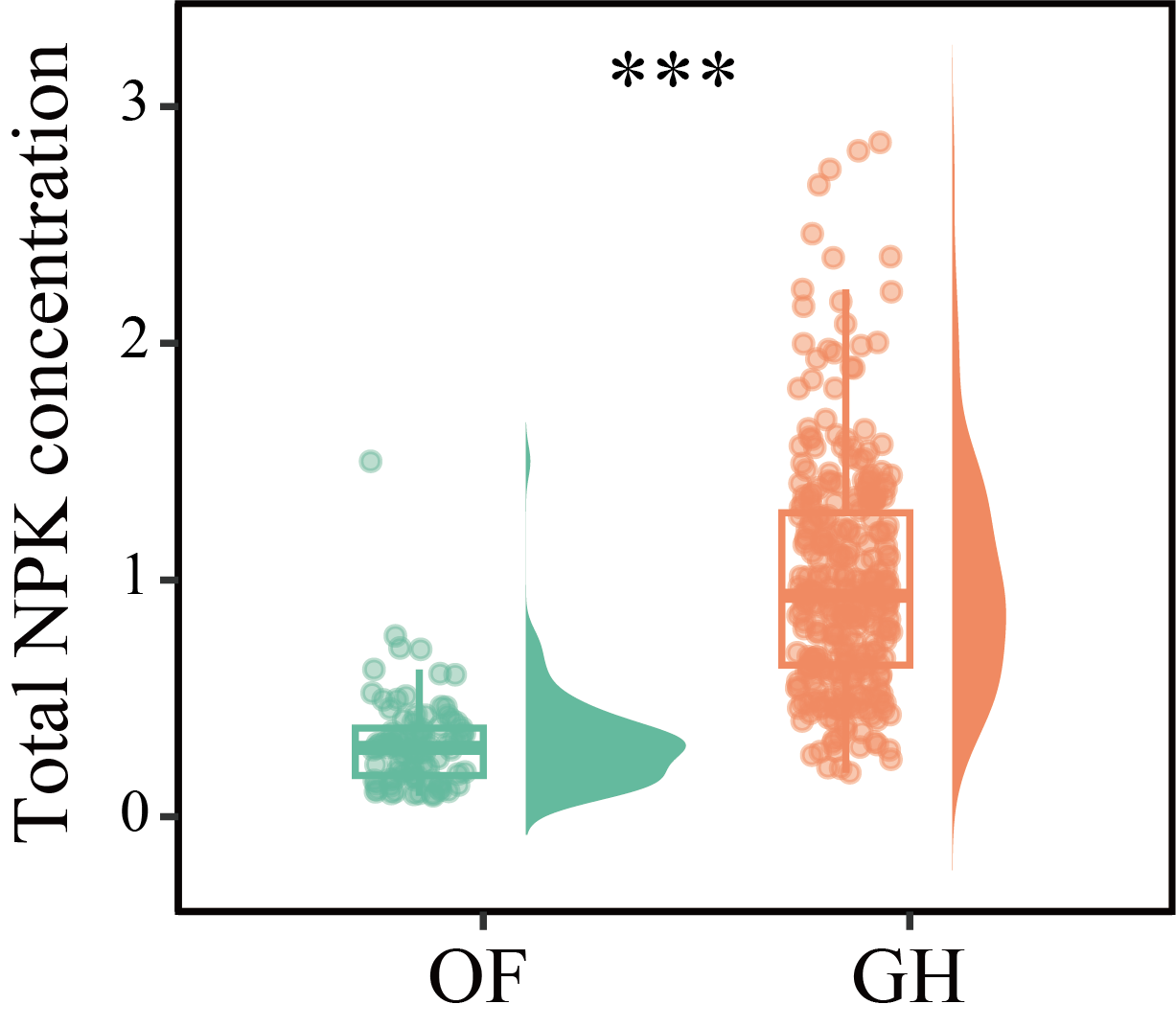
**

**Figure S3**. Difference in total NPK concentration between the open field (OF) and greenhouse (GH) soils (Wilcoxon rank-sum test; ***, *p* < 0.001). The total NPK concentrations were calculated by summing the standardized concentrations of NH_4_^+^-N, NO_3_^−^-N, AP, and AK.


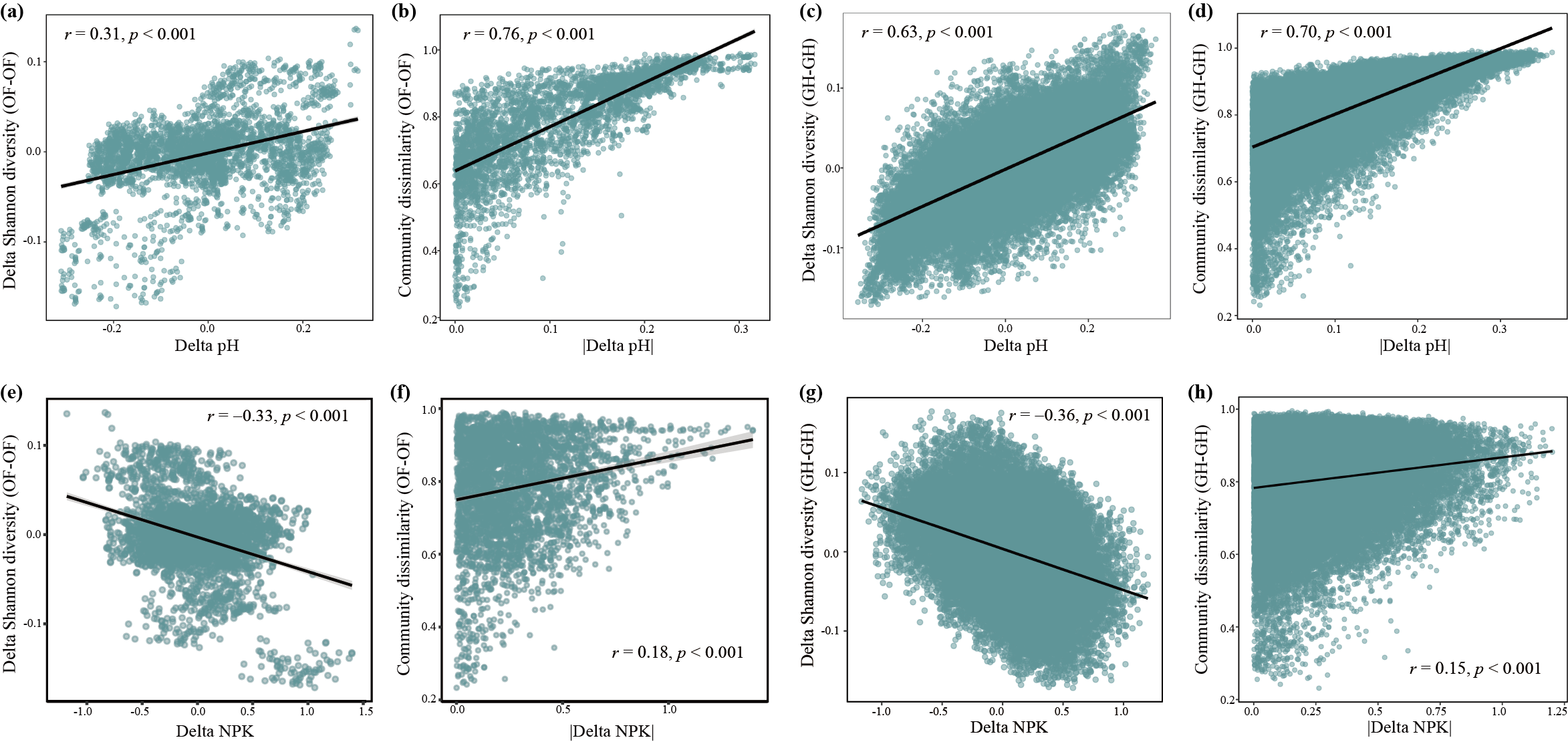


**Figure S4**. Relationship between differences in bacterial communities and delta soil pH and NPK concentration in open field (OF) or greenhouse (GH) soils (*n* = 396). **a** and **c** Relationships between delta Shannon diversity and delta pH concentration in OF (**a**) or GH (**c**) soils. **b** and **d** Relationships between community dissimilarity and |delta pH | concentration in OF (**b**) or GH (**d**) soils. **e** and **g** Relationships between delta Shannon diversity and delta NPK concentration in OF (**e**) or GH (**g**) soils. **f** and **h** Relationships between community dissimilarity and |delta NPK| concentration in OF (**f**) or GH (**h**) soils. Delta is calculated from the logarithm-transformed (log10) ratio of all possible OF or GH pairs. The black lines represent ordinary least squares linear regressions. The gray areas represent the 95% confidence intervals.


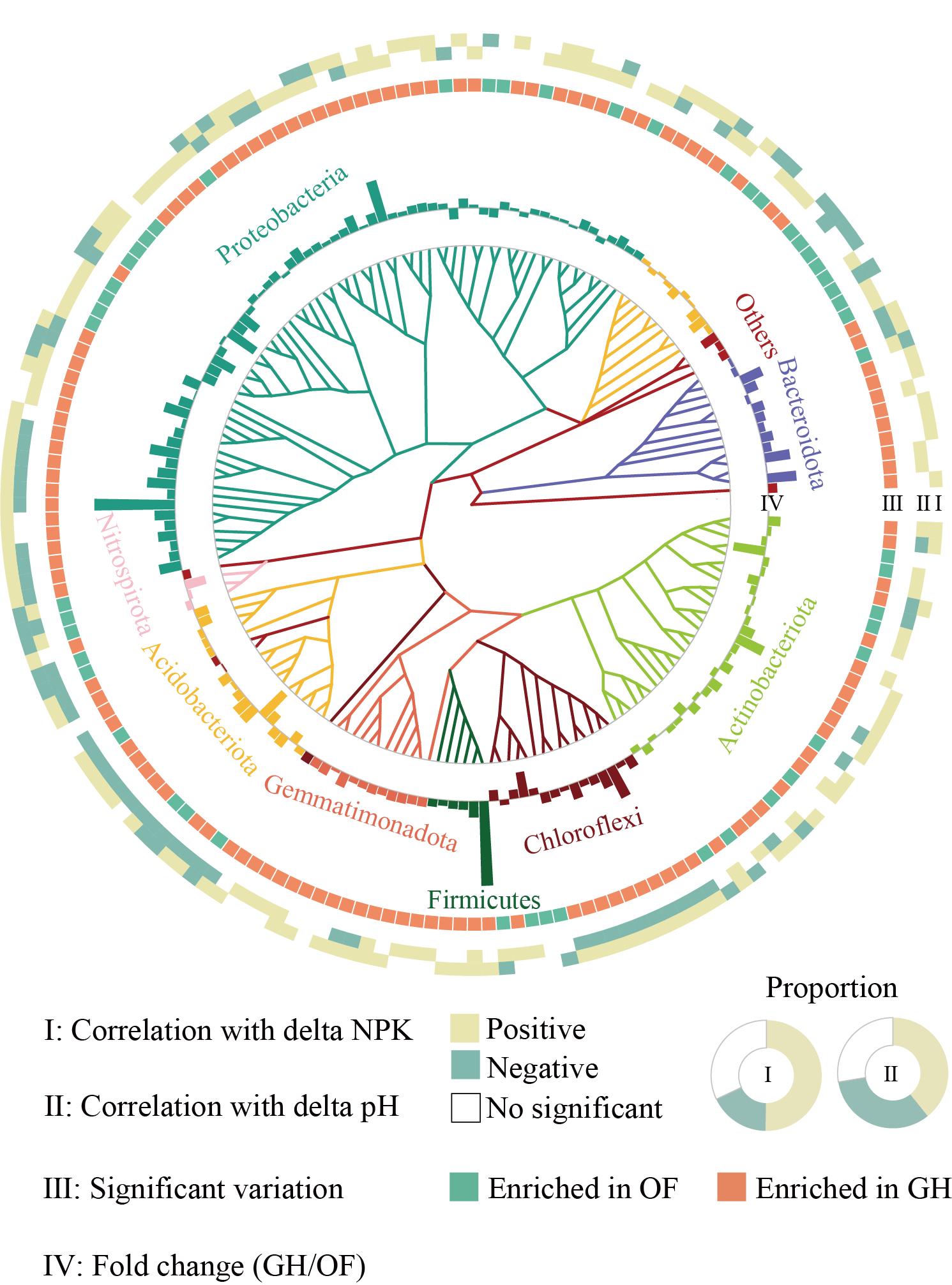


**Figure S5**. Phylogenetic tree of bacterial zOTUs significantly (FDR adjusted *p* < 0.05) enriched in the OF or GH soils based on the Wilcoxon rank-sum test. The zOTUs with average relative abundances >0.05% in the OF and GH soils were retained in the figure. Taxonomic information, zOTU enrichment, the difference in the relative abundances of zOTUs between the OF and GH soils, the relationships between the delta relative abundances of the zOTUs, delta pH, and NPK concentration are displayed from the inner to the outer circles. Pie plots show the ratios of zOTUs correlated with delta pH and NPK concentration.

**
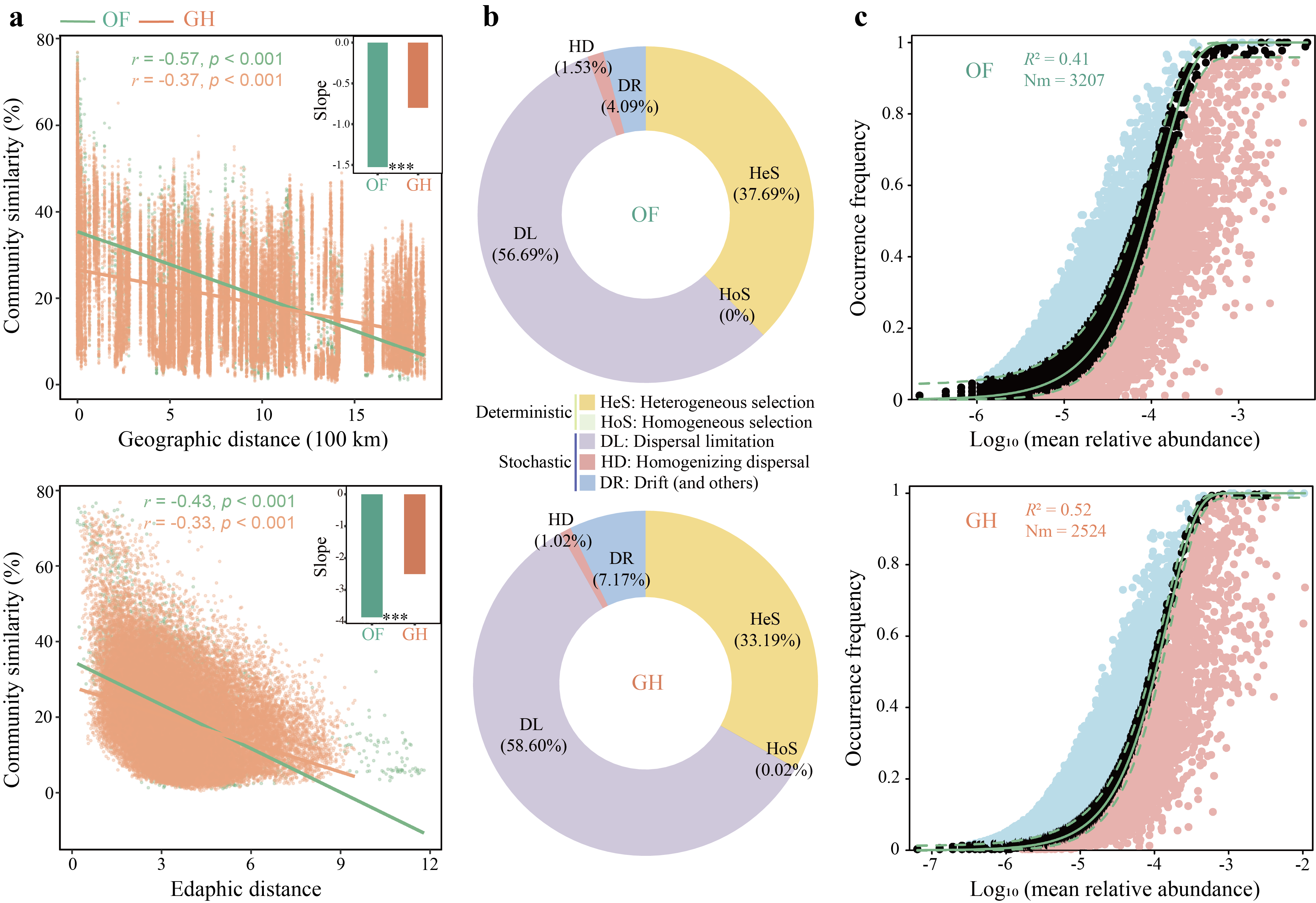
Figure S6**. Assembly mechanisms of the bacterial communities in the open field (OF) and greenhouse (GH) soils (*n* = 396). **a** Distance-decay relationships between bacterial-community similarity, geographic distance, and edaphic distance in the OF (*n* = 89) and GH (*n* = 307) soils. **b** Relative importance of deterministic and stochastic processes mediating the assembly of the bacterial communities in the OF and GH soils. **c** Fit of the neutral community model (NCM) of the bacterial communities in the OF and GH soils. The solid lines indicate the best fit to the NCM, and the dashed lines represent 95% confidence intervals of the model prediction. Bacterial communities that occur more or less frequently than predicted are shown in different colors. Nm indicates the metacommunity size times immigration, and *R*^2^ indicates the fit to this model.


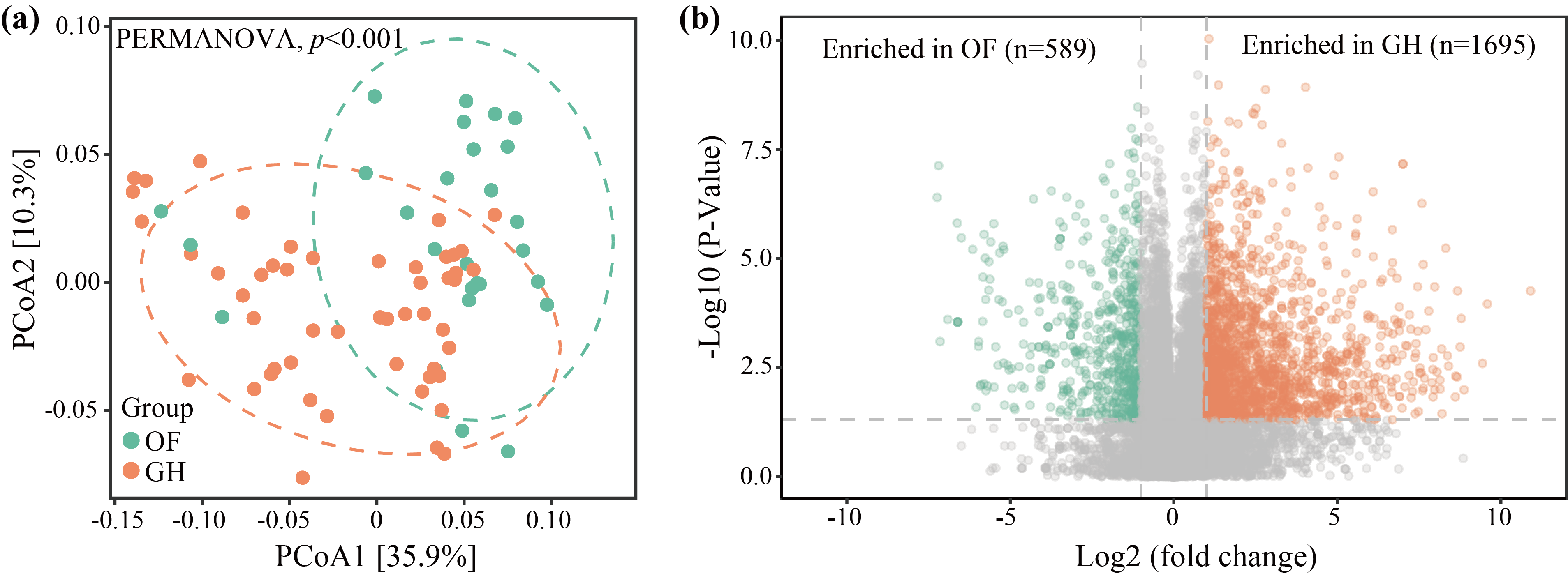


**Figure S7**. Genes in the Kyoto Encyclopedia of Genes and Genomes (KEGG) Ontology (KOs) in the open field (OF) and greenhouse (GH) soils (*n* = 78). **a** Principal coordinate analysis (PCoA) of Bray-Curtis dissimilarity based on KO count tables for each sample showing differences in the functional composition between the OF and GH soils. **b** Difference in the abundances of KOs between the OF and GH soils. Green and orange circles indicate the KOs significantly (|log2 fold change| > 1 and FDR adjusted *p* < 0.05) enriched in the OF and GH soils, respectively.


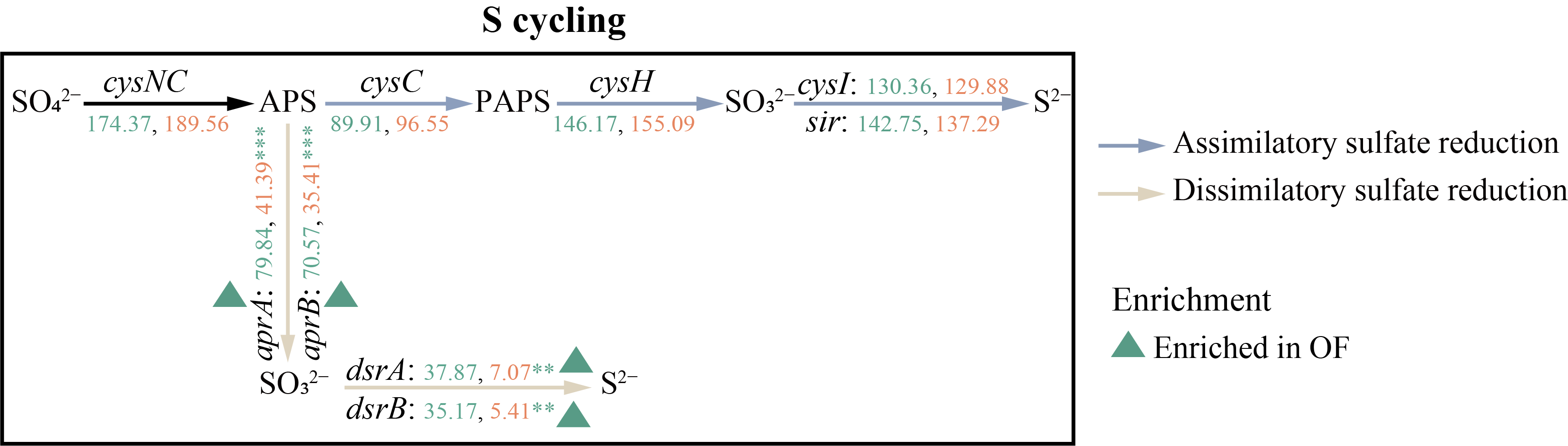


**Figure S8.** Differences in the abundances (normalized counts per million) of functional genes involved in sulfur (S) cycling between the open field (OF) and greenhouse (GH) soils (*n* = 78) (Wilcoxon rank-sum test; **, FDR adjusted *p* < 0.01; ***, FDR adjusted *p* < 0.001).


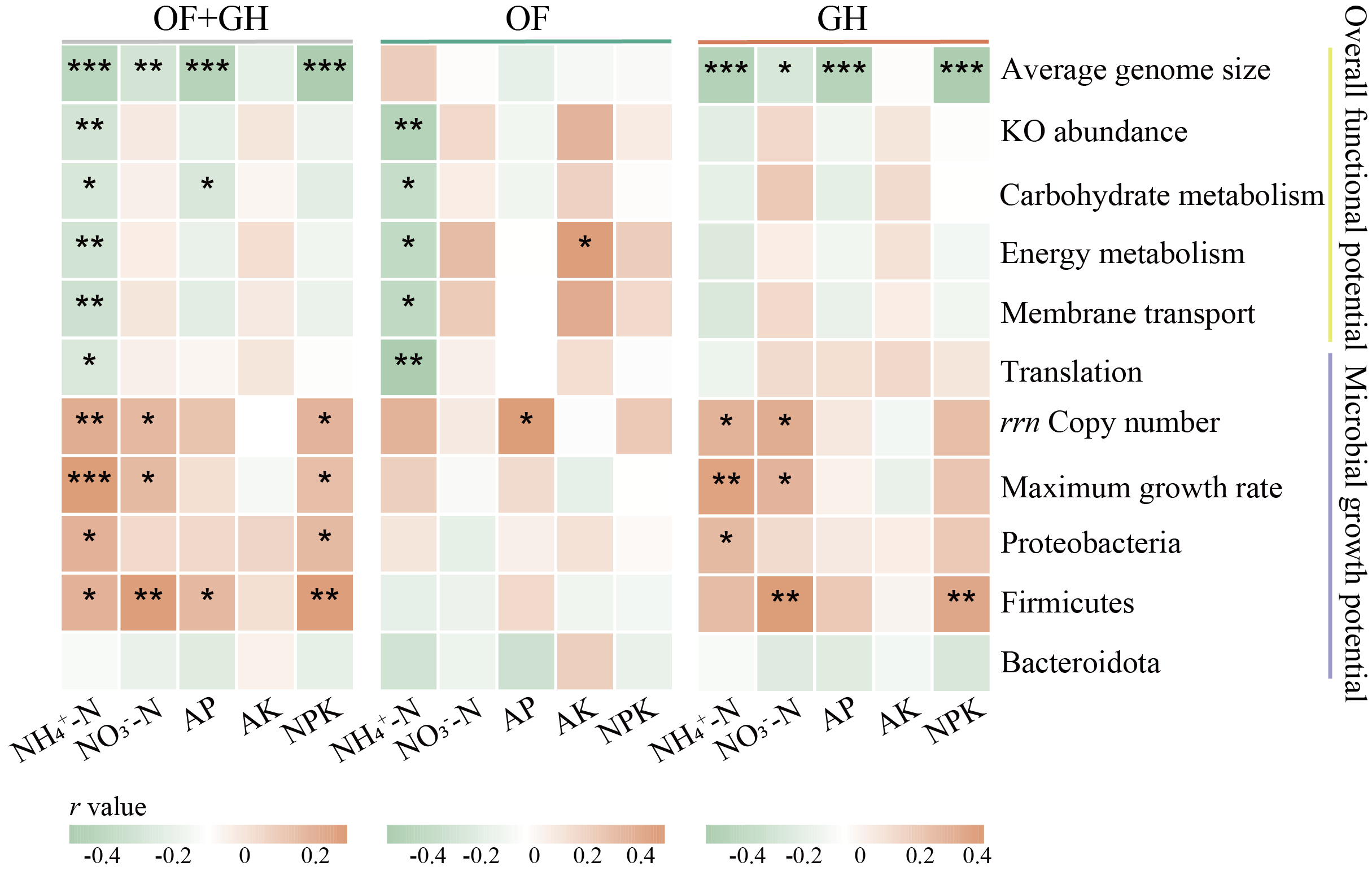


**Figure S9.** Heatmap showing the correlations between soil nutrient concentration and microbial functional potential and growth potential (*n* = 78). The color denotes the correlation coefficient determined by Pearson’s correlation. Asterisks indicate significant correlations (*, *p* < 0.05; **, *p* < 0.01; ***, *p* < 0.001).


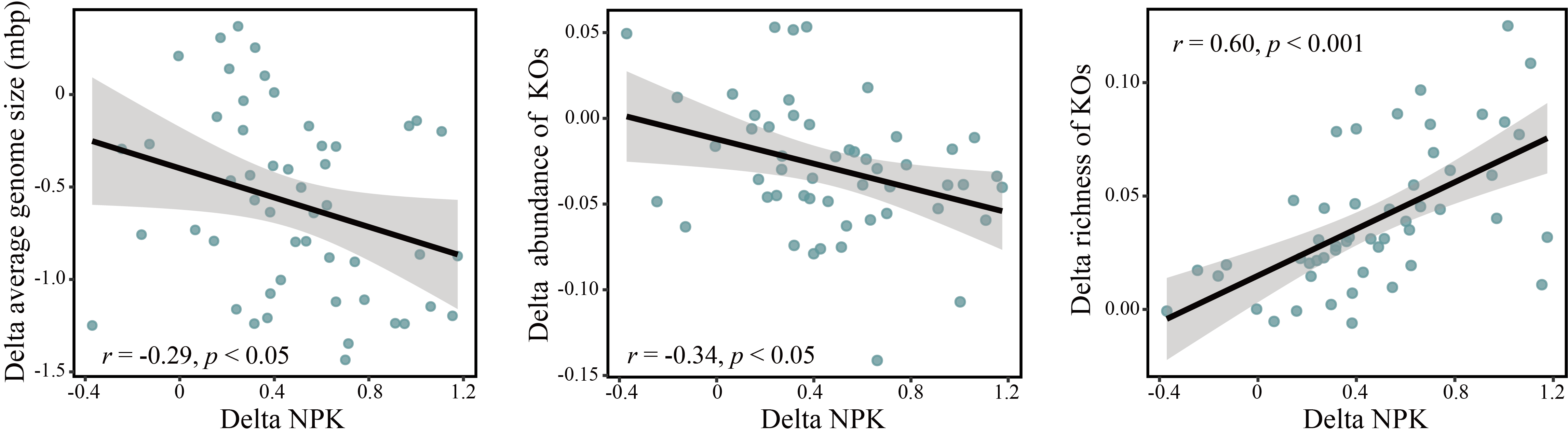


**Figure S10**. Relationships between delta average genome size, abundance of KOs, richness of KOs, and delta NPK concentration (*n* = 78). The delta value was calculated as the log_10_-transformed ratio derived from pairwise comparisons between the GH and OF soils. The black lines represent ordinary least squares linear regressions. The gray areas represent the 95% confidence intervals.


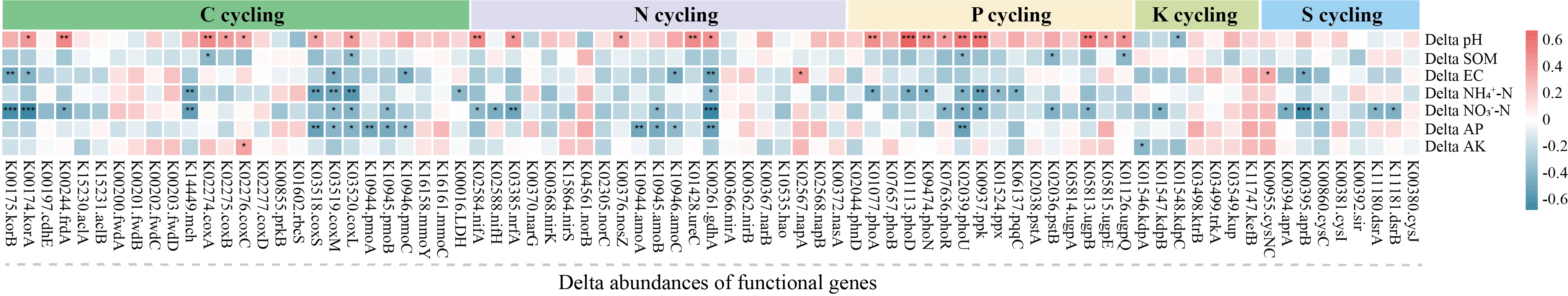


**Figure S11.** Heatmap of the correlations between delta (greenhouse–open field) soil properties and abundances of functional genes involved in the cycling of carbon (C), nitrogen (N), phosphorus (P), and potassium (K), and sulfur (S) (*n* = 78). EC, soil electrical conductivity; SOM, soil organic matter; AP, available phosphorus; AK, available potassium; NH_4_^+^-N, ammonium nitrogen; NO_3_^−^-N, nitrate nitrogen. *, ** and *** represent FDR adjusted *p* < 0.05, < 0.01 and < 0.001, respectively.


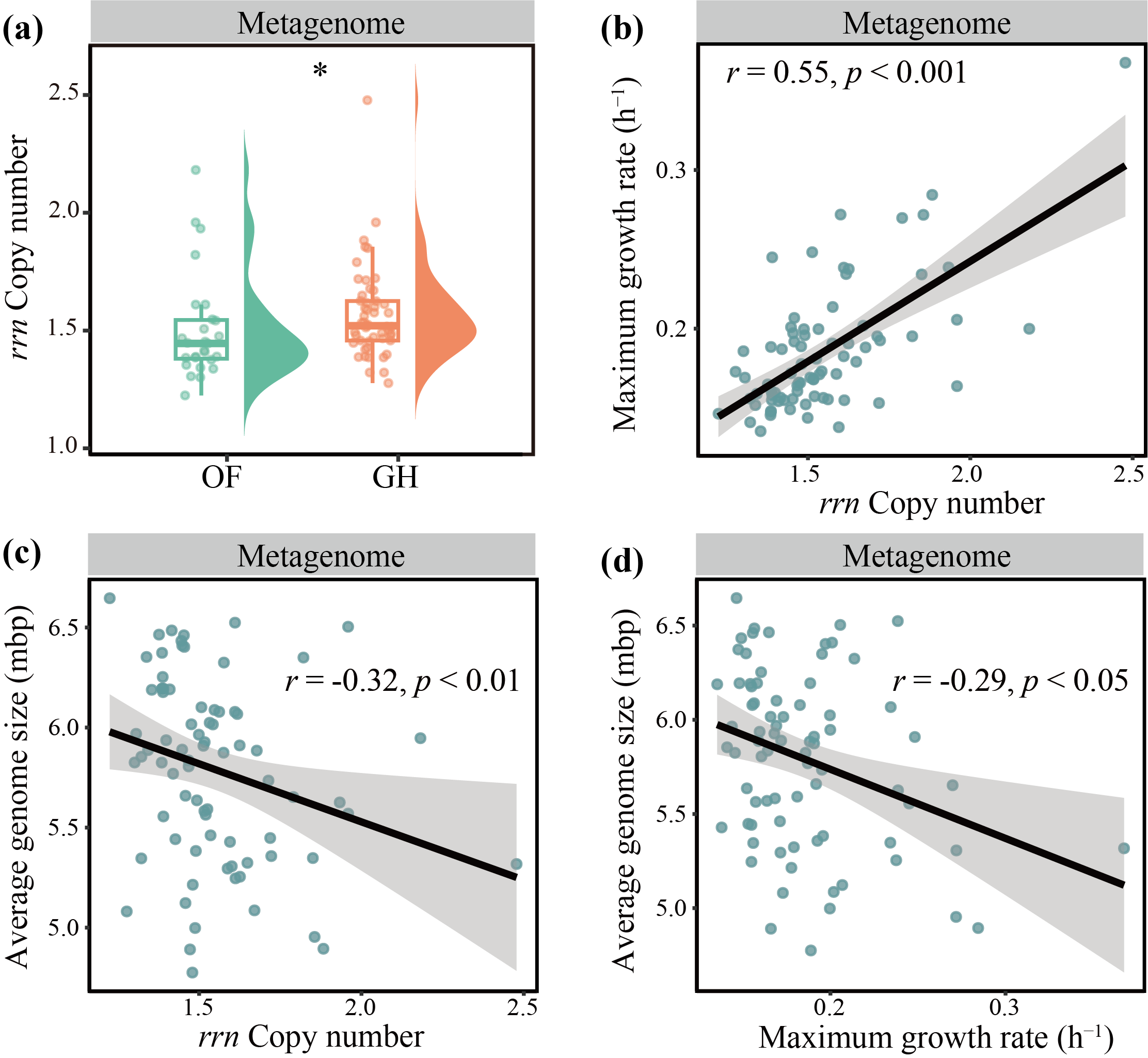


**Figure S12**. Differences in *rrn* copy number estimated from metagenomic contigs, and the relationships among *rrn* copy number, maximum growth rate (metagenomic contigs), and average genome size (metagenomic contigs) in the open field (OF) and greenhouse (GH) soils (*n* = 78). **a** Difference in *rrn* copy number between the OF and GH soils (Wilcoxon rank-sum test, * *p* < 0.05). **b** Relationships between maximum growth rate and *rrn* copy number. **c** and **d** Relationships between average genome size, *rrn* copy number, and maximum growth rate. The black lines represent ordinary least squares linear regressions. The gray areas represent the 95% confidence intervals.


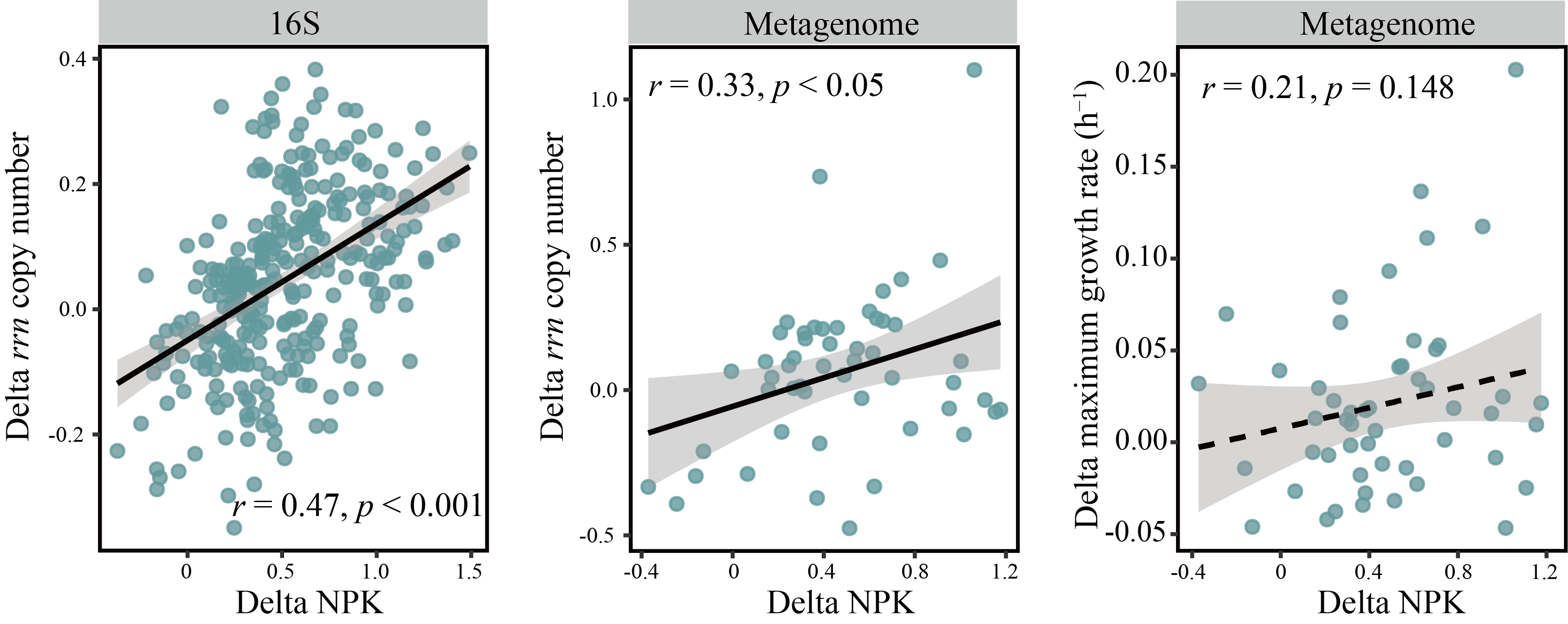


**Figure S13.** Correlations between delta bacterial life-history strategies and delta NPK concentration. The delta value was calculated as the log_10_-transformed ratio derived from pairwise comparisons between the greenhouse and open field soils. The black lines represent ordinary least squares linear regressions. The gray areas represent the 95% confidence intervals.


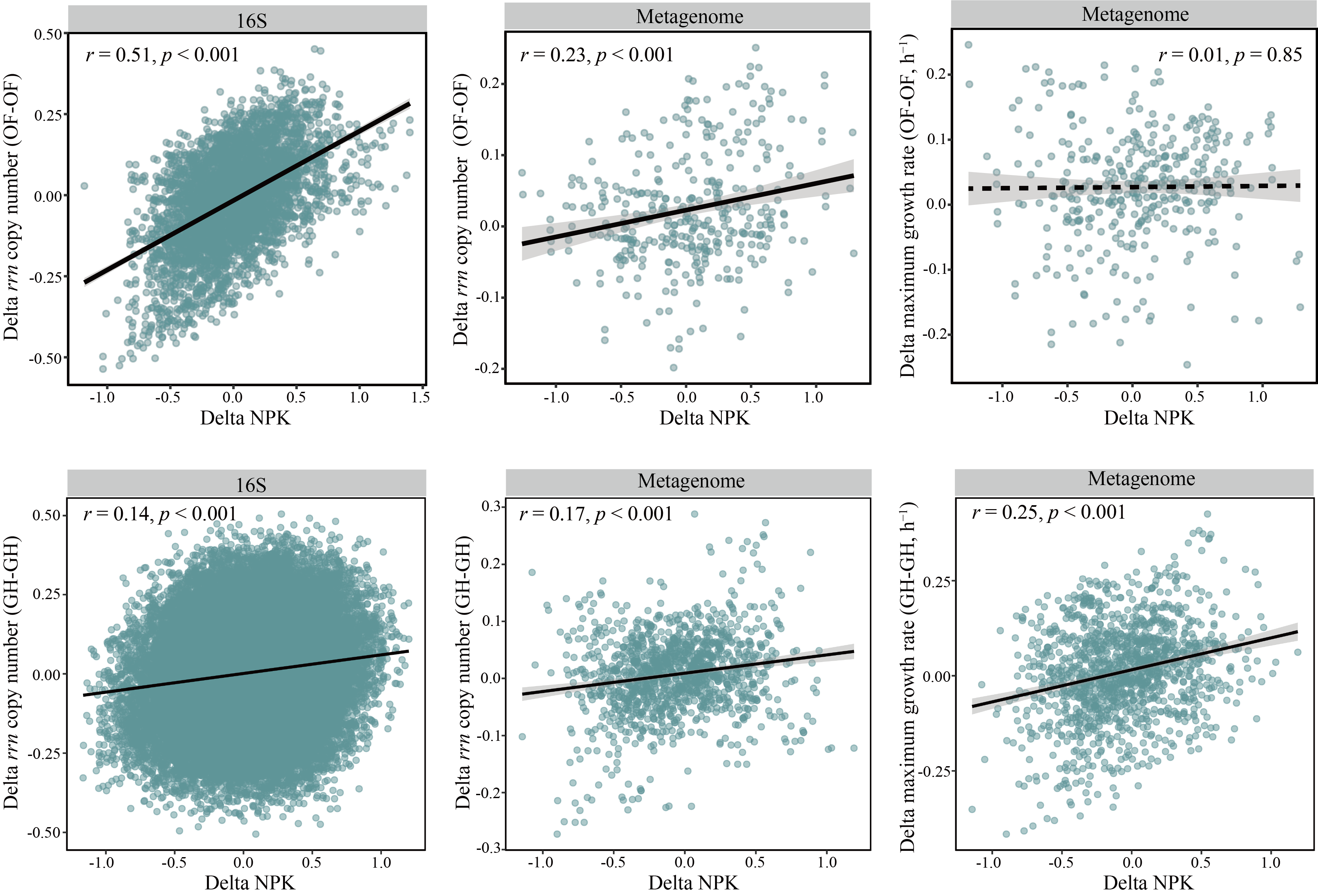


**Figure S14.** Correlations between delta bacterial life-history strategies and delta NPK concentration in open field (OF) or greenhouse (GH) soils. The delta value was calculated as the log_10_-transformed ratio derived from pairwise comparisons between the open field or greenhouse soils. The black lines represent ordinary least squares linear regressions. The gray areas represent the 95% confidence intervals.


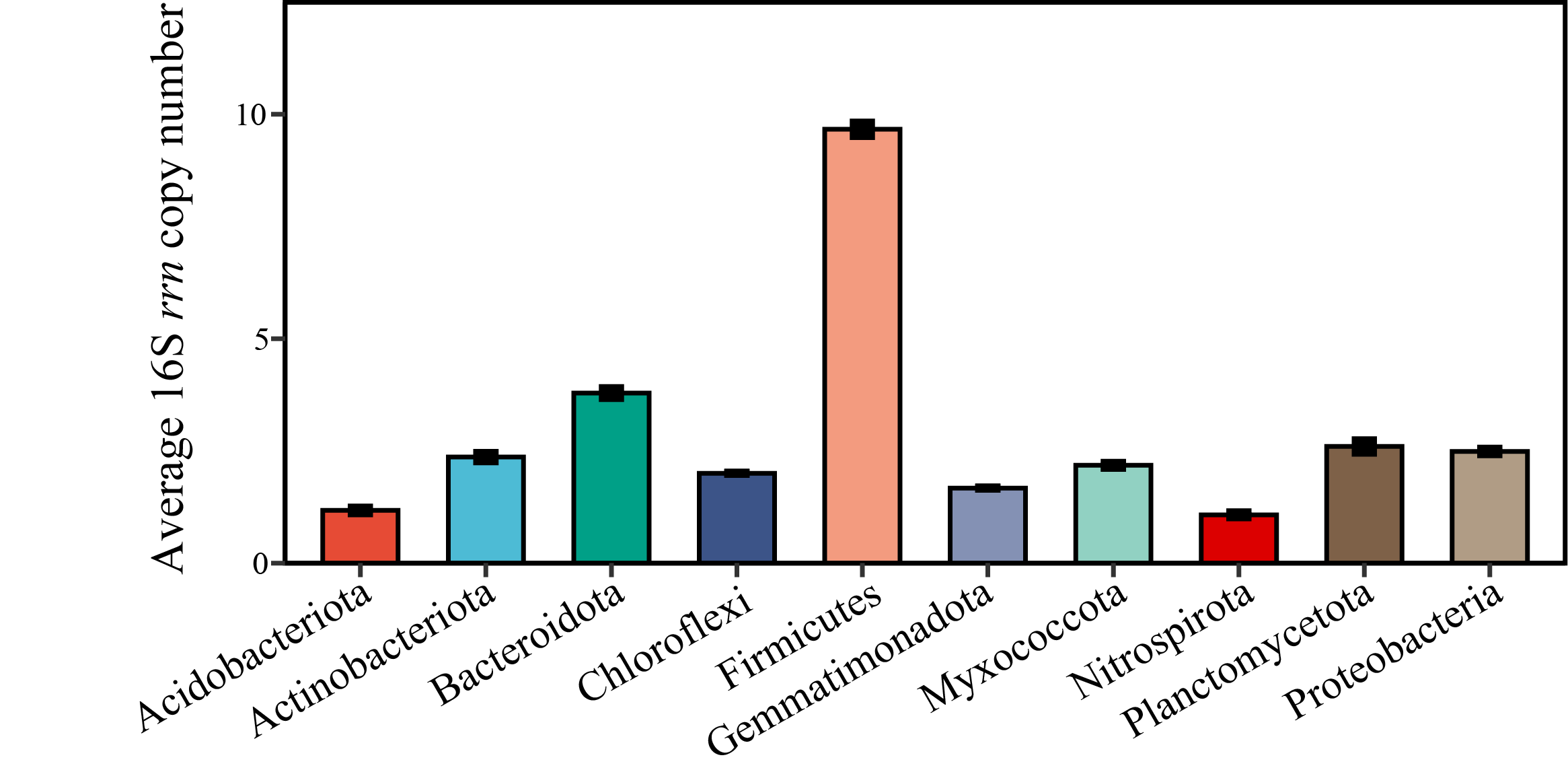


**Figure S15.** The average estimated 16S *rrn* copy number of zOTUs derived from each phylum (*n* = 396). Error bars indicate standard errors.


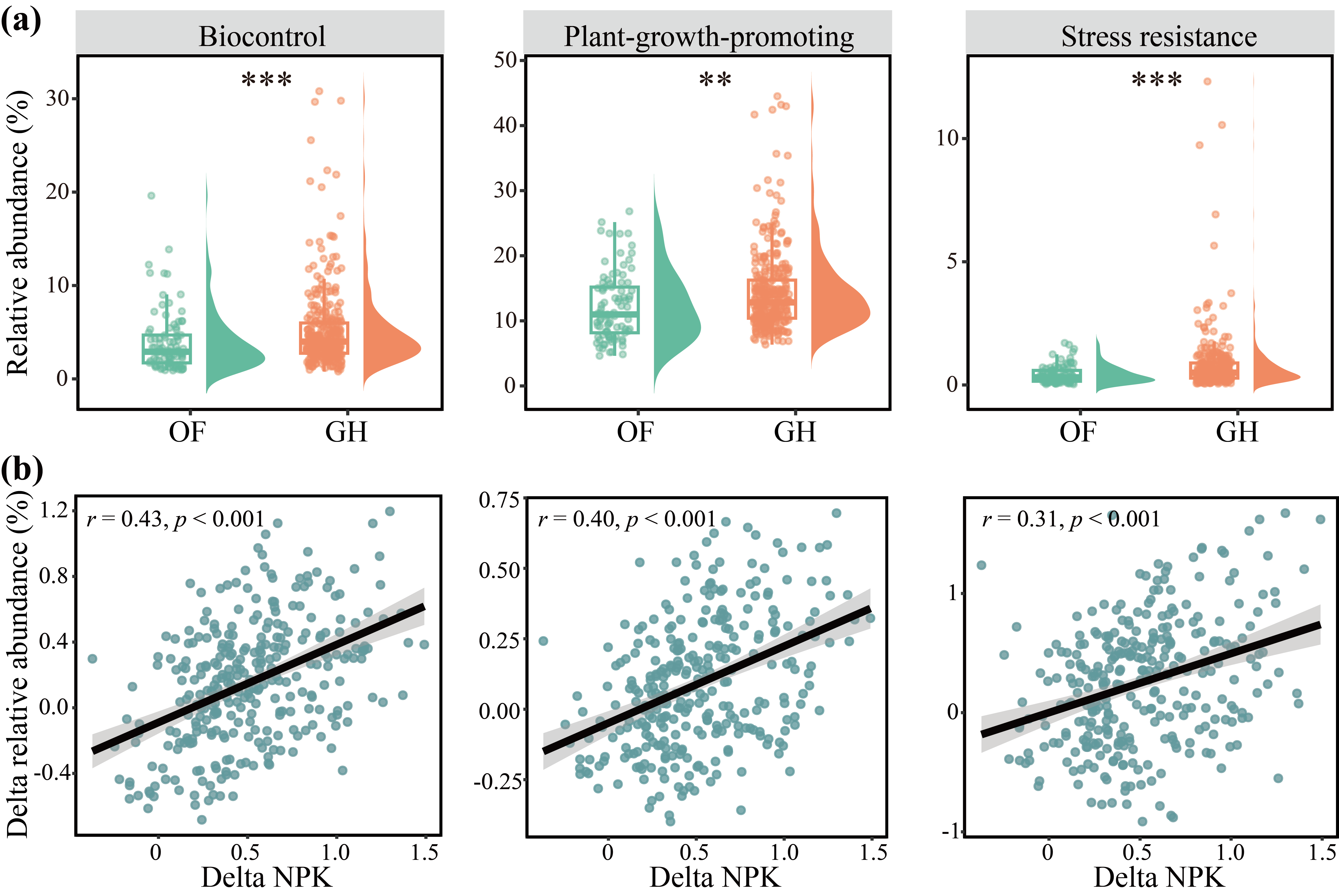


**Figure S16.** Differences in the relative abundance of plant-beneficial bacteria (PBB) based on 16S amplicon data and its relationships with NPK concentration in the open field (OF) and greenhouse (GH) soils (*n* = 396). **a** Differences in the relative abundance of PBB, including biocontrol agents, bacteria promoting plant growth, and stress-resistant bacteria (Wilcoxon rank-sum test; **, *p* < 0.01; ***, *p* < 0.001). **b** Relationships between the delta relative abundance of PBB and delta NPK concentration. The delta value was calculated as the log_10_-transformed ratio derived from pairwise comparisons between the GH and OF soils. The black lines represent ordinary least squares linear regressions. The gray areas represent the 95% confidence intervals.


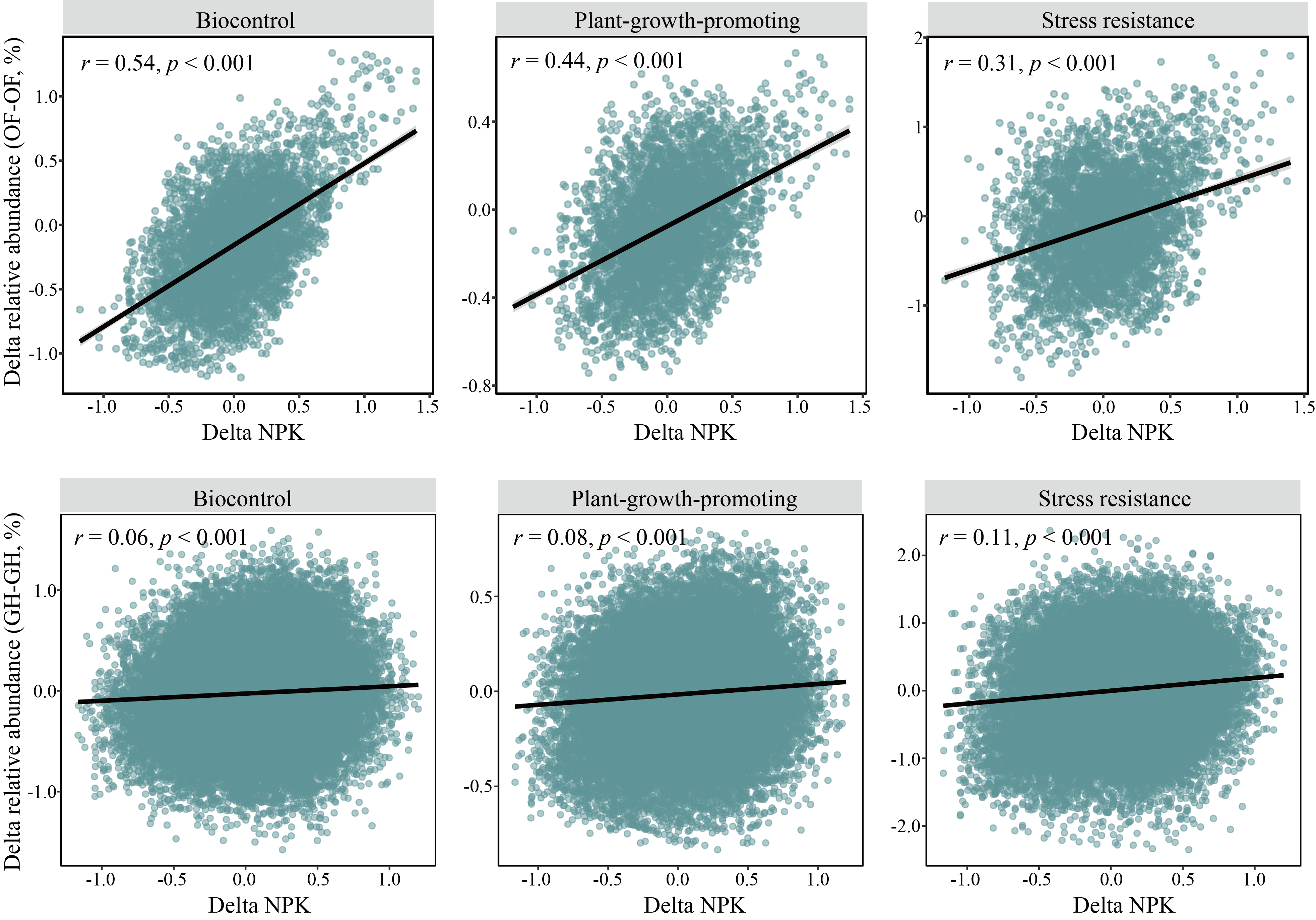


**Figure S17.** Differences in the relative abundance of plant-beneficial bacteria (PBB) based on 16S amplicon data and its relationships with NPK concentration in the open field (OF) or greenhouse (GH) soils (*n* = 396). The delta value was calculated as the log_10_-transformed ratio derived from pairwise comparisons between the OF or GH soils. The black lines represent ordinary least squares linear regressions. The gray areas represent the 95% confidence intervals.


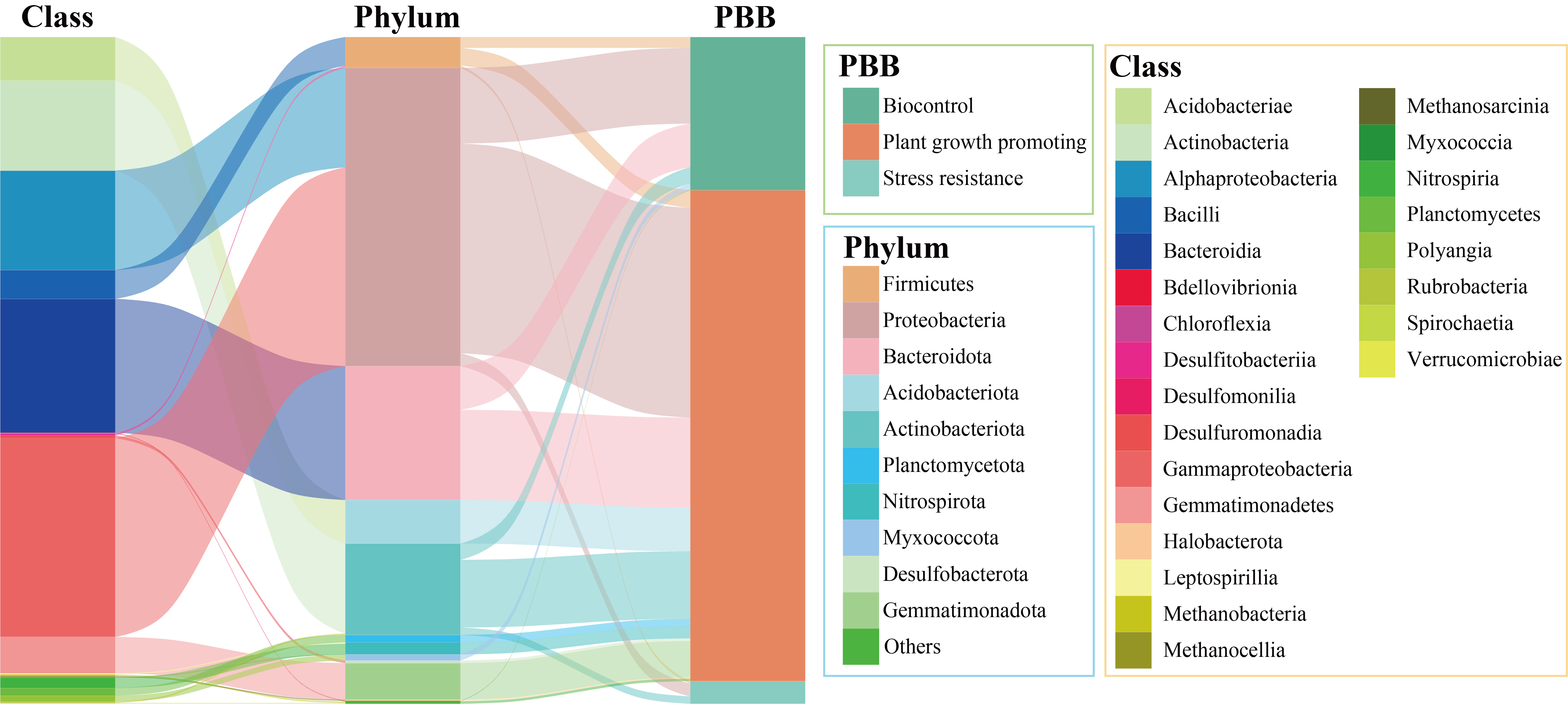


**Figure S18.** Alluvial diagrams of the proportions of bacterial phyla and classes annotated as plant-beneficial bacteria (PBB, *n* = 396).


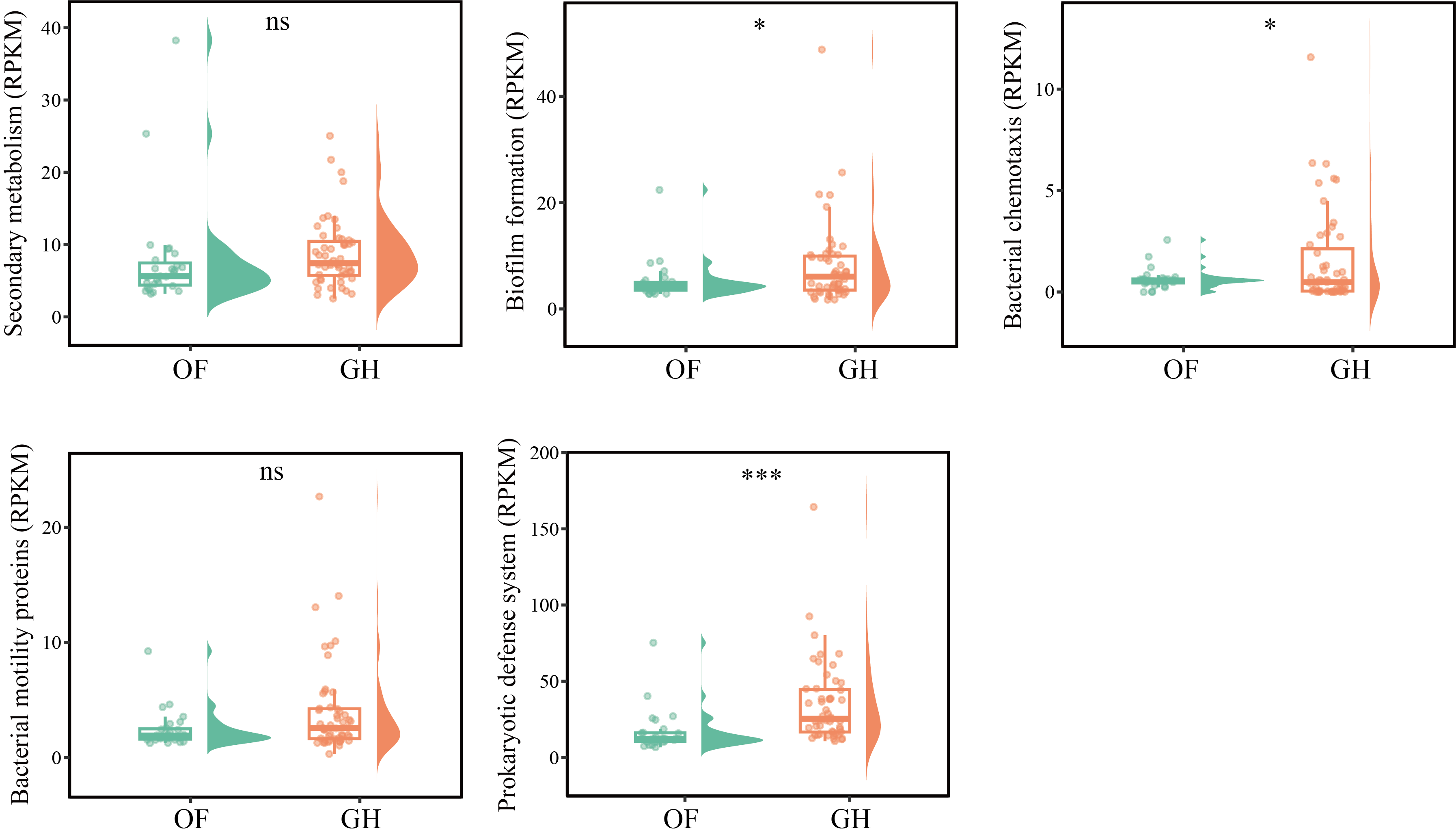


**Figure S19.** Differences in the abundances of secondary metabolism, biofilm formation, bacterial chemotaxis, bacterial motility proteins, and prokaryotic defensive systems of MAGs between the open field (OF) and greenhouse (GH) soils (Wilcoxon rank-sum test; ns, not significant, *, *p* < 0.05; ***, *p* < 0.001).


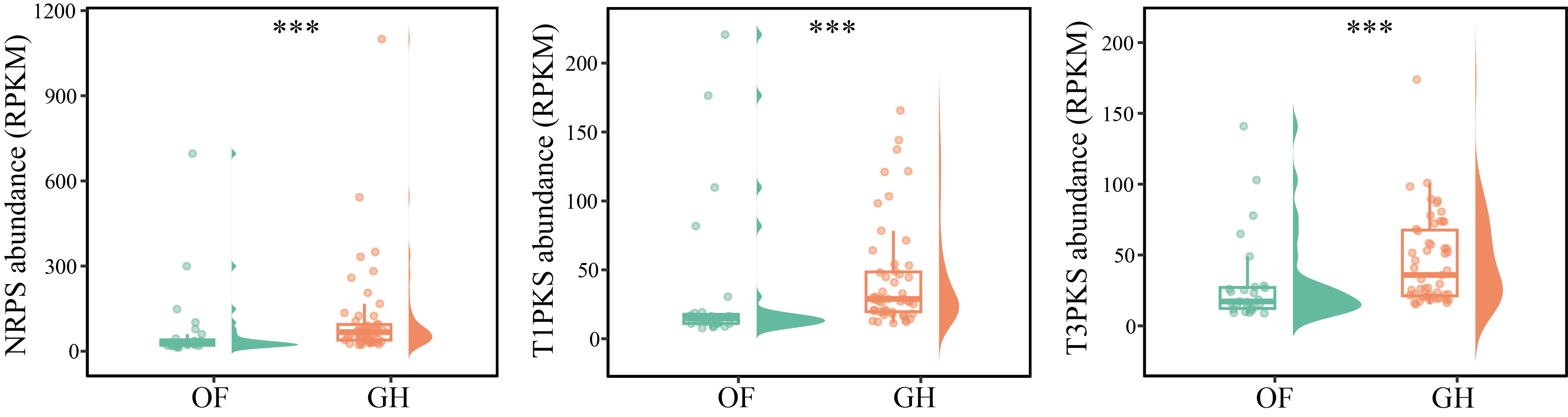


**Figure S20.** Differences in the abundances of biosynthetic gene clusters in MAGs between the open field (OF) and greenhouse (GH) soils, including nonribosomal peptide synthetase (NRPS), type I polyketide synthase (T1PKS), and type III polyketide synthase (T3PKS) (Wilcoxon rank-sum test; ***, *p* < 0.001).

**Table. S1** Summary of quality-filtered reads.

| Soil environment | Sample ID | Number of 16S reads | Number of metagenomic reads |
| --- | --- | --- | --- |
| Greenhouse soil | BB_1 | 92,164 | N |
| Greenhouse soil | BB_10 | 106,222 | N |
| Greenhouse soil | BB_11 | 79,071 | N |
| Greenhouse soil | BB_12 | 119,353 | 40,062,719 |
| Greenhouse soil | BB_13 | 124,047 | N |
| Greenhouse soil | BB_14 | 122,082 | N |
| Greenhouse soil | BB_15 | 117,062 | N |
| Greenhouse soil | BB_16 | 110,581 | N |
| Greenhouse soil | BB_17 | 109,327 | N |
| Greenhouse soil | BB_18 | 129,759 | N |
| Greenhouse soil | BB_19 | 109,831 | N |
| Greenhouse soil | BB_2 | 110,156 | N |
| Greenhouse soil | BB_20 | 140,573 | N |
| Greenhouse soil | BB_21 | 133,502 | N |
| Greenhouse soil | BB_22 | 119,893 | 41,312,511 |
| Greenhouse soil | BB_23 | 103,110 | 43,154,489 |
| Greenhouse soil | BB_3 | 109,817 | N |
| Greenhouse soil | BB_4 | 90,607 | N |
| Greenhouse soil | BB_5 | 111,279 | N |
| Greenhouse soil | BB_6 | 121,359 | 32,651,710 |
| Greenhouse soil | BB_7 | 129,974 | 38,531,110 |
| Greenhouse soil | BB_8 | 137,104 | 41,472,538 |
| Greenhouse soil | BB_9 | 120,161 | N |
| Open field soil | BB_D1 | 103,794 | N |
| Open field soil | BB_D2 | 109,188 | 38,060,270 |
| Open field soil | BB_D3 | 132,009 | 59,145,912 |
| Open field soil | BB_D4 | 117,692 | N |
| Open field soil | BB_D5 | 83,359 | N |
| Open field soil | BB_D6 | 110,084 | 47,345,983 |
| Greenhouse soil | DG_10 | 116,151 | N |
| Greenhouse soil | DG_11 | 122,441 | N |
| Greenhouse soil | DG_12 | 135,320 | N |
| Greenhouse soil | DG_6 | 109,283 | N |
| Greenhouse soil | DG_7 | 94,348 | N |
| Greenhouse soil | DG_9 | 131,074 | N |
| Open field soil | DG_D1 | 102,928 | N |
| Open field soil | DG_D2 | 112,155 | N |
| Greenhouse soil | FS_3 | 96,918 | N |
| Greenhouse soil | FS_4 | 107,302 | N |
| Greenhouse soil | FS_5 | 116,229 | N |
| Greenhouse soil | FS_6 | 118,436 | N |
| Open field soil | FS_D2 | 108,073 | N |
| Greenhouse soil | FZ_1 | 133,757 | 40,444,629 |
| Greenhouse soil | FZ_10 | 120,851 | N |
| Greenhouse soil | FZ_11 | 109,841 | N |
| Greenhouse soil | FZ_12 | 109,755 | 39,535,560 |
| Greenhouse soil | FZ_13 | 131,542 | N |
| Greenhouse soil | FZ_14 | 124,176 | N |
| Greenhouse soil | FZ_15 | 115,367 | N |
| Greenhouse soil | FZ_16 | 131,454 | N |
| Greenhouse soil | FZ_17 | 115,775 | N |
| Greenhouse soil | FZ_18 | 111,977 | N |
| Greenhouse soil | FZ_19 | 112,482 | N |
| Greenhouse soil | FZ_2 | 126,944 | N |
| Greenhouse soil | FZ_20 | 89,432 | 38,711,274 |
| Greenhouse soil | FZ_21 | 101,174 | N |
| Greenhouse soil | FZ_22 | 89,904 | N |
| Greenhouse soil | FZ_23 | 109,455 | N |
| Greenhouse soil | FZ_24 | 101,893 | N |
| Greenhouse soil | FZ_25 | 109,091 | N |
| Greenhouse soil | FZ_26 | 110,042 | N |
| Greenhouse soil | FZ_27 | 121,820 | N |
| Greenhouse soil | FZ_3 | 137,235 | N |
| Greenhouse soil | FZ_4 | 143,769 | N |
| Greenhouse soil | FZ_5 | 145,161 | N |
| Greenhouse soil | FZ_6 | 115,517 | N |
| Greenhouse soil | FZ_7 | 120,840 | N |
| Greenhouse soil | FZ_8 | 118,582 | N |
| Greenhouse soil | FZ_9 | 112,146 | N |
| Open field soil | FZ_D1 | 112,766 | 38,134,995 |
| Open field soil | FZ_D2 | 96,785 | 34,982,166 |
| Open field soil | FZ_D3 | 68,974 | 43,599,124 |
| Open field soil | FZ_D4 | 112,381 | N |
| Open field soil | FZ_D5 | 119,854 | N |
| Open field soil | FZ_D6 | 128,074 | N |
| Greenhouse soil | GZ_1 | 183,748 | N |
| Greenhouse soil | GZ_2 | 104,541 | 37,746,986 |
| Greenhouse soil | GZ_3 | 84,256 | N |
| Greenhouse soil | GZ_4 | 86,707 | N |
| Greenhouse soil | GZ_5 | 125,889 | N |
| Greenhouse soil | GZ_6 | 116,045 | 37,379,503 |
| Greenhouse soil | GZ_7 | 127,462 | N |
| Greenhouse soil | GZ_8 | 124,759 | N |
| Greenhouse soil | GZ_9 | 119,282 | N |
| Open field soil | GZ_D1 | 114,997 | 44,094,127 |
| Open field soil | GZ_D2 | 91,605 | N |
| Greenhouse soil | HZ_1 | 120,557 | N |
| Greenhouse soil | HZ_10 | 158,297 | N |
| Greenhouse soil | HZ_2 | 114,951 | N |
| Greenhouse soil | HZ_3 | 110,574 | N |
| Greenhouse soil | HZ_6 | 95,190 | N |
| Greenhouse soil | HZ_7 | 108,043 | N |
| Greenhouse soil | HZ_8 | 101,273 | N |
| Greenhouse soil | HZ_9 | 145,696 | N |
| Open field soil | HZ_D1 | 120,031 | N |
| Open field soil | HZ_D2 | 102,012 | N |
| Open field soil | HZ_D3 | 97,961 | N |
| Greenhouse soil | JM_1 | 112,621 | N |
| Greenhouse soil | JM_2 | 103,088 | N |
| Greenhouse soil | JM_3 | 137,245 | N |
| Greenhouse soil | JM_5 | 113,137 | N |
| Greenhouse soil | JM_6 | 114,134 | N |
| Greenhouse soil | JM_7 | 113,817 | N |
| Open field soil | JM_D1 | 107,711 | N |
| Open field soil | JM_D2 | 100,466 | N |
| Greenhouse soil | JY_1 | 93,943 | N |
| Greenhouse soil | JY_2 | 91,913 | N |
| Greenhouse soil | JY_3 | 115,085 | N |
| Greenhouse soil | JY_4 | 106,915 | N |
| Greenhouse soil | JY_5 | 117,930 | N |
| Greenhouse soil | JY_6 | 116,171 | N |
| Open field soil | JY_D1 | 267,859 | N |
| Open field soil | JY_D2 | 105,965 | N |
| Greenhouse soil | MZ_1 | 98,587 | 40,252,717 |
| Greenhouse soil | MZ_10 | 113,722 | N |
| Greenhouse soil | MZ_2 | 113,918 | N |
| Greenhouse soil | MZ_3 | 110,681 | 35,815,627 |
| Greenhouse soil | MZ_4 | 123,708 | N |
| Greenhouse soil | MZ_6 | 110,254 | N |
| Greenhouse soil | MZ_7 | 94,181 | N |
| Greenhouse soil | MZ_8 | 114,468 | N |
| Greenhouse soil | MZ_9 | 109,553 | N |
| Open field soil | MZ_D1 | 116,642 | 38,763,235 |
| Open field soil | MZ_D2 | 88,146 | N |
| Open field soil | MZ_D3 | 90,886 | N |
| Greenhouse soil | NX_1 | 71,095 | N |
| Greenhouse soil | NX_10 | 66,259 | N |
| Greenhouse soil | NX_11 | 65,250 | N |
| Greenhouse soil | NX_12 | 67,801 | 39,631,731 |
| Greenhouse soil | NX_13 | 67,056 | N |
| Greenhouse soil | NX_14 | 82,703 | N |
| Greenhouse soil | NX_15 | 82,699 | N |
| Greenhouse soil | NX_16 | 79,686 | N |
| Greenhouse soil | NX_17 | 79,406 | N |
| Greenhouse soil | NX_18 | 85,683 | N |
| Greenhouse soil | NX_19 | 79,206 | N |
| Greenhouse soil | NX_2 | 72,760 | 42,004,081 |
| Greenhouse soil | NX_20 | 84,443 | N |
| Greenhouse soil | NX_21 | 78,158 | N |
| Greenhouse soil | NX_22 | 81,390 | N |
| Greenhouse soil | NX_23 | 78,657 | N |
| Greenhouse soil | NX_24 | 81,987 | 38,343,897 |
| Greenhouse soil | NX_25 | 80,701 | N |
| Greenhouse soil | NX_26 | 80,615 | N |
| Greenhouse soil | NX_27 | 82,928 | 37,093,095 |
| Greenhouse soil | NX_28 | 80,616 | N |
| Greenhouse soil | NX_29 | 73,100 | N |
| Greenhouse soil | NX_3 | 72,037 | N |
| Greenhouse soil | NX_30 | 69,245 | N |
| Greenhouse soil | NX_31 | 90,648 | N |
| Greenhouse soil | NX_32 | 79,101 | N |
| Greenhouse soil | NX_33 | 81,030 | N |
| Greenhouse soil | NX_34 | 77,275 | N |
| Greenhouse soil | NX_35 | 72,108 | N |
| Greenhouse soil | NX_36 | 81,370 | N |
| Greenhouse soil | NX_37 | 74,650 | N |
| Greenhouse soil | NX_38 | 75,244 | N |
| Greenhouse soil | NX_39 | 69,796 | N |
| Greenhouse soil | NX_4 | 68,738 | N |
| Greenhouse soil | NX_40 | 75,496 | N |
| Greenhouse soil | NX_41 | 97,241 | N |
| Greenhouse soil | NX_42 | 87,244 | N |
| Greenhouse soil | NX_43 | 87,338 | N |
| Greenhouse soil | NX_44 | 96,835 | N |
| Greenhouse soil | NX_45 | 83,212 | N |
| Greenhouse soil | NX_46 | 83,178 | N |
| Greenhouse soil | NX_47 | 91,324 | N |
| Greenhouse soil | NX_48 | 104,346 | N |
| Greenhouse soil | NX_49 | 100,257 | N |
| Greenhouse soil | NX_5 | 73,926 | N |
| Greenhouse soil | NX_50 | 89,458 | N |
| Greenhouse soil | NX_51 | 55,347 | N |
| Greenhouse soil | NX_52 | 78,129 | N |
| Greenhouse soil | NX_6 | 78,296 | 48,637,825 |
| Greenhouse soil | NX_7 | 82,952 | N |
| Greenhouse soil | NX_8 | 68,583 | N |
| Greenhouse soil | NX_9 | 79,017 | 42,835,239 |
| Open field soil | NX_D1 | 92,465 | 45,649,220 |
| Open field soil | NX_D10 | 96,952 | N |
| Open field soil | NX_D11 | 94,659 | N |
| Open field soil | NX_D12 | 101,162 | N |
| Open field soil | NX_D13 | 104,274 | N |
| Open field soil | NX_D14 | 90,598 | N |
| Open field soil | NX_D15 | 96,925 | N |
| Open field soil | NX_D2 | 80,314 | N |
| Open field soil | NX_D3 | 80,845 | 38,471,112 |
| Open field soil | NX_D4 | 84,914 | N |
| Open field soil | NX_D5 | 83,745 | N |
| Open field soil | NX_D6 | 88,441 | 35,414,823 |
| Open field soil | NX_D7 | 80,391 | N |
| Open field soil | NX_D8 | 91,452 | N |
| Open field soil | NX_D9 | 82,689 | N |
| Greenhouse soil | SG_1 | 69,964 | 36,671,316 |
| Greenhouse soil | SG_10 | 75,372 | N |
| Greenhouse soil | SG_11 | 71,054 | N |
| Greenhouse soil | SG_12 | 75,864 | N |
| Greenhouse soil | SG_13 | 86,958 | N |
| Greenhouse soil | SG_14 | 73,065 | N |
| Greenhouse soil | SG_15 | 74,360 | N |
| Greenhouse soil | SG_16 | 67,761 | 39,457,293 |
| Greenhouse soil | SG_17 | 67,234 | N |
| Greenhouse soil | SG_18 | 78,529 | N |
| Greenhouse soil | SG_19 | 73,757 | N |
| Greenhouse soil | SG_2 | 73,802 | N |
| Greenhouse soil | SG_20 | 76,690 | 35,295,106 |
| Greenhouse soil | SG_21 | 72,972 | N |
| Greenhouse soil | SG_22 | 72,646 | N |
| Greenhouse soil | SG_23 | 67,970 | N |
| Greenhouse soil | SG_24 | 72,769 | N |
| Greenhouse soil | SG_25 | 66,747 | N |
| Greenhouse soil | SG_26 | 74,521 | N |
| Greenhouse soil | SG_27 | 77,261 | 33,833,357 |
| Greenhouse soil | SG_28 | 79,699 | N |
| Greenhouse soil | SG_29 | 78,241 | N |
| Greenhouse soil | SG_3 | 77,226 | 38,254,543 |
| Greenhouse soil | SG_30 | 95,863 | 33,776,537 |
| Greenhouse soil | SG_31 | 73,495 | N |
| Greenhouse soil | SG_32 | 79,940 | N |
| Greenhouse soil | SG_33 | 90,965 | N |
| Greenhouse soil | SG_34 | 75,148 | N |
| Greenhouse soil | SG_35 | 91,807 | N |
| Greenhouse soil | SG_36 | 82,845 | N |
| Greenhouse soil | SG_37 | 66,360 | N |
| Greenhouse soil | SG_4 | 73,640 | N |
| Greenhouse soil | SG_5 | 73,058 | N |
| Greenhouse soil | SG_6 | 73,732 | N |
| Greenhouse soil | SG_7 | 77,787 | N |
| Greenhouse soil | SG_8 | 73,437 | N |
| Greenhouse soil | SG_9 | 74,326 | N |
| Open field soil | SG_D1 | 71,173 | 41,771,811 |
| Open field soil | SG_D10 | 81,818 | N |
| Open field soil | SG_D2 | 71,645 | N |
| Open field soil | SG_D3 | 68,881 | N |
| Open field soil | SG_D4 | 69,785 | 34,956,267 |
| Open field soil | SG_D5 | 74,271 | N |
| Open field soil | SG_D6 | 88,939 | N |
| Open field soil | SG_D7 | 83,184 | 34,484,020 |
| Open field soil | SG_D8 | 79,311 | N |
| Open field soil | SG_D9 | 80,137 | N |
| Greenhouse soil | ST_1 | 126,301 | N |
| Greenhouse soil | ST_2 | 120,855 | N |
| Greenhouse soil | ST_3 | 112,657 | N |
| Greenhouse soil | ST_4 | 123,026 | N |
| Greenhouse soil | ST_5 | 111,963 | N |
| Greenhouse soil | ST_6 | 105,262 | N |
| Open field soil | ST_D1 | 122,517 | N |
| Open field soil | ST_D2 | 101,941 | N |
| Greenhouse soil | SW_4 | 124,386 | 39,364,315 |
| Greenhouse soil | SW_5 | 128,970 | N |
| Greenhouse soil | SW_6 | 101,142 | N |
| Greenhouse soil | SW_7 | 79,636 | N |
| Greenhouse soil | SW_8 | 133,792 | 43,952,550 |
| Greenhouse soil | SW_9 | 118,684 | N |
| Open field soil | SW_D1 | 107,001 | 41,454,077 |
| Greenhouse soil | WZ_1 | 84,690 | N |
| Greenhouse soil | WZ_10 | 84,540 | N |
| Greenhouse soil | WZ_11 | 82,301 | N |
| Greenhouse soil | WZ_12 | 87,574 | N |
| Greenhouse soil | WZ_13 | 89,445 | N |
| Greenhouse soil | WZ_14 | 89,191 | N |
| Greenhouse soil | WZ_15 | 95,104 | N |
| Greenhouse soil | WZ_16 | 91,976 | 36,572,856 |
| Greenhouse soil | WZ_17 | 85,230 | N |
| Greenhouse soil | WZ_18 | 90,456 | 39,508,506 |
| Greenhouse soil | WZ_19 | 101,685 | N |
| Greenhouse soil | WZ_2 | 82,851 | 36,379,489 |
| Greenhouse soil | WZ_20 | 91,397 | N |
| Greenhouse soil | WZ_21 | 91,967 | N |
| Greenhouse soil | WZ_22 | 95,662 | N |
| Greenhouse soil | WZ_23 | 97,155 | N |
| Greenhouse soil | WZ_24 | 75,489 | N |
| Greenhouse soil | WZ_25 | 73,547 | N |
| Greenhouse soil | WZ_26 | 72,099 | N |
| Greenhouse soil | WZ_27 | 77,877 | N |
| Greenhouse soil | WZ_28 | 80,981 | N |
| Greenhouse soil | WZ_29 | 76,486 | N |
| Greenhouse soil | WZ_3 | 86,432 | N |
| Greenhouse soil | WZ_30 | 84,376 | 33,543,195 |
| Greenhouse soil | WZ_31 | 83,385 | N |
| Greenhouse soil | WZ_32 | 74,682 | 36,163,115 |
| Greenhouse soil | WZ_33 | 78,758 | N |
| Greenhouse soil | WZ_34 | 81,015 | N |
| Greenhouse soil | WZ_35 | 57,482 | N |
| Greenhouse soil | WZ_36 | 77,212 | N |
| Greenhouse soil | WZ_37 | 72,953 | N |
| Greenhouse soil | WZ_38 | 71,517 | N |
| Greenhouse soil | WZ_4 | 105,331 | N |
| Greenhouse soil | WZ_5 | 82,086 | N |
| Greenhouse soil | WZ_6 | 88,203 | 33,071,858 |
| Greenhouse soil | WZ_7 | 88,881 | N |
| Greenhouse soil | WZ_8 | 90,024 | N |
| Greenhouse soil | WZ_9 | 84,755 | N |
| Open field soil | WZ_D1 | 81,420 | N |
| Open field soil | WZ_D2 | 78,951 | 35,863,191 |
| Open field soil | WZ_D3 | 82,743 | N |
| Open field soil | WZ_D4 | 86,164 | 40,617,585 |
| Open field soil | WZ_D5 | 99,520 | N |
| Open field soil | WZ_D6 | 104,934 | N |
| Open field soil | WZ_D7 | 69,501 | 34,594,037 |
| Open field soil | WZ_D8 | 73,232 | N |
| Open field soil | WZ_D9 | 68,941 | N |
| Greenhouse soil | YC_1 | 93,588 | N |
| Greenhouse soil | YC_10 | 91,465 | N |
| Greenhouse soil | YC_11 | 66,565 | N |
| Greenhouse soil | YC_12 | 82,909 | N |
| Greenhouse soil | YC_13 | 92,363 | N |
| Greenhouse soil | YC_14 | 82,071 | N |
| Greenhouse soil | YC_15 | 87,881 | 39,088,033 |
| Greenhouse soil | YC_16 | 93,086 | N |
| Greenhouse soil | YC_17 | 80,053 | N |
| Greenhouse soil | YC_18 | 83,703 | 35,966,939 |
| Greenhouse soil | YC_19 | 71,703 | N |
| Greenhouse soil | YC_2 | 80,211 | 37,061,633 |
| Greenhouse soil | YC_20 | 67,406 | 38,809,049 |
| Greenhouse soil | YC_21 | 84,588 | N |
| Greenhouse soil | YC_22 | 86,851 | N |
| Greenhouse soil | YC_23 | 99,469 | N |
| Greenhouse soil | YC_24 | 111,118 | N |
| Greenhouse soil | YC_25 | 138,596 | 40,679,789 |
| Greenhouse soil | YC_26 | 112,741 | N |
| Greenhouse soil | YC_3 | 88,773 | N |
| Greenhouse soil | YC_4 | 96,131 | N |
| Greenhouse soil | YC_5 | 83,202 | N |
| Greenhouse soil | YC_6 | 94,163 | 35,045,396 |
| Greenhouse soil | YC_7 | 73,365 | N |
| Greenhouse soil | YC_8 | 84,896 | N |
| Greenhouse soil | YC_9 | 84,042 | N |
| Open field soil | YC_D1 | 117,780 | 39,967,260 |
| Open field soil | YC_D2 | 121,610 | N |
| Open field soil | YC_D3 | 107,213 | N |
| Open field soil | YC_D4 | 77,077 | N |
| Open field soil | YC_D5 | 96,382 | 33,831,747 |
| Open field soil | YC_D6 | 100,198 | N |
| Open field soil | YC_D7 | 99,449 | N |
| Open field soil | YC_D8 | 98,934 | 40,502,325 |
| Open field soil | YC_D9 | 89,462 | N |
| Greenhouse soil | YF_1 | 89,798 | N |
| Open field soil | YF_D1 | 108,632 | N |
| Greenhouse soil | YZ_1 | 83,376 | 32,369,097 |
| Greenhouse soil | YZ_10 | 79,945 | N |
| Greenhouse soil | YZ_11 | 108,154 | N |
| Greenhouse soil | YZ_12 | 74,138 | N |
| Greenhouse soil | YZ_13 | 73,162 | 51,144,879 |
| Greenhouse soil | YZ_14 | 65,652 | 44,033,951 |
| Greenhouse soil | YZ_15 | 87,109 | 37,011,269 |
| Greenhouse soil | YZ_16 | 90,351 | 47,459,587 |
| Greenhouse soil | YZ_17 | 96,796 | N |
| Greenhouse soil | YZ_18 | 90,852 | N |
| Greenhouse soil | YZ_19 | 88,948 | N |
| Greenhouse soil | YZ_2 | 80,981 | 41,560,470 |
| Greenhouse soil | YZ_3 | 100,852 | N |
| Greenhouse soil | YZ_4 | 82,485 | N |
| Greenhouse soil | YZ_5 | 83,905 | N |
| Greenhouse soil | YZ_6 | 108,620 | N |
| Greenhouse soil | YZ_7 | 77,729 | N |
| Greenhouse soil | YZ_8 | 56,228 | N |
| Greenhouse soil | YZ_9 | 90,155 | N |
| Open field soil | YZ_D1 | 84,477 | 43,230,139 |
| Open field soil | YZ_D2 | 65,370 | N |
| Open field soil | YZ_D3 | 77,639 | N |
| Open field soil | YZ_D4 | 86,682 | N |
| Open field soil | YZ_D5 | 84,167 | 48,896,006 |
| Open field soil | YZ_D6 | 57,156 | 68,948,343 |
| Open field soil | YZ_D7 | 79,029 | N |
| Greenhouse soil | ZMD_1 | 78,369 | 33,357,038 |
| Greenhouse soil | ZMD_10 | 80,075 | N |
| Greenhouse soil | ZMD_11 | 85,272 | N |
| Greenhouse soil | ZMD_12 | 85,354 | N |
| Greenhouse soil | ZMD_13 | 86,287 | 35,045,915 |
| Greenhouse soil | ZMD_14 | 85,177 | N |
| Greenhouse soil | ZMD_15 | 79,839 | 36,306,239 |
| Greenhouse soil | ZMD_16 | 91,495 | N |
| Greenhouse soil | ZMD_17 | 97,141 | 37,374,250 |
| Greenhouse soil | ZMD_18 | 90,247 | N |
| Greenhouse soil | ZMD_19 | 85,753 | N |
| Greenhouse soil | ZMD_2 | 99,155 | N |
| Greenhouse soil | ZMD_20 | 88,659 | 34,938,656 |
| Greenhouse soil | ZMD_21 | 88,329 | N |
| Greenhouse soil | ZMD_22 | 86,040 | N |
| Greenhouse soil | ZMD_23 | 88,483 | N |
| Greenhouse soil | ZMD_24 | 81,983 | N |
| Greenhouse soil | ZMD_3 | 84,979 | N |
| Greenhouse soil | ZMD_4 | 85,214 | N |
| Greenhouse soil | ZMD_5 | 87,757 | N |
| Greenhouse soil | ZMD_6 | 106,090 | 33,836,118 |
| Greenhouse soil | ZMD_7 | 87,620 | N |
| Greenhouse soil | ZMD_8 | 89,396 | N |
| Greenhouse soil | ZMD_9 | 80,997 | N |
| Open field soil | ZMD_D1 | 83,844 | 36,984,446 |
| Open field soil | ZMD_D2 | 102,288 | N |
| Open field soil | ZMD_D3 | 80,872 | N |
| Open field soil | ZMD_D4 | 83,678 | N |
| Open field soil | ZMD_D5 | 93,640 | 37,750,251 |
| Open field soil | ZMD_D6 | 81,728 | N |
| Open field soil | ZMD_D7 | 80,177 | 39,849,257 |
| Open field soil | ZMD_D8 | 123,919 | N |

N indicates that the sample was not subjected to metagenomic sequencing.

**Table. S2** Copiotrophic and oligotrophic bacterial phyla were defined in the study.

| **Phylum** | **Oligotroph or** **Copiotroph** | **References** |
| --- | --- | --- |
| Actinobacteriota | Oligotroph | (Aira, Perez-Losada, & Dominguez, 2019; Li et al., 2021; Zhou, Wang, Jiang, & Luo, 2017) |
| Proteobacteria | Copiotroph | (Chen et al., 2022; Li et al., 2021; Stone et al., 2023) |
| Acidobacteriota | Oligotroph | (Chen et al., 2022; Fierer, Bradford, & Jackson, 2007; Fierer et al., 2012; Li et al., 2021; Li et al., 2014; Männistö, Ganzert, Tiirola, Häggblom, & Stark, 2016; Stone et al., 2023; Zhou et al., 2017) |
| Chloroflexi | Oligotroph | (Chen et al., 2022; Li et al., 2021; Stone et al., 2023) |
| Bacteroidota | Copiotroph | (Chen et al., 2022; Fierer et al., 2007; Fierer et al., 2012; Männistö et al., 2016; Stone et al., 2023; Zhou et al., 2017) |
| Gemmatimonadota | Oligotroph | (Cederlund et al., 2014; Chen et al., 2022; Li et al., 2021) |
| Planctomycetota | Oligotroph | (Li et al., 2021; Männistö et al., 2016; Stone et al., 2023) |
| Firmicutes | Copiotroph | (Chen et al., 2022; Francioli et al., 2016; Li et al., 2021; Stone et al., 2023) |
| Myxococcota | Oligotroph | (Li et al., 2023) |
| Nitrospirota | Oligotroph | (van Kessel et al., 2015; Daims et al., 2015) |

**References**

Aira, M., Perez-Losada, M., & Dominguez, J. (2019). Microbiome dynamics during cast ageing in the earthworm Aporrectodea caliginosa. Appl Soil Ecol, 139, 56-63.

Cederlund, H., Wessen, E., Enwall, K., Jones, C. M., Juhanson, J., Pell, M., Philippot L., Hallin, S. (2014). Soil carbon quality and nitrogen fertilization structure bacterial communities with predictable responses of major bacterial phyla. Appl Soil Ecol, 84, 62-68.

Chen, H., Jing, Q., Liu, X., Zhou, X., Fang, C., Li, B., Zhou S., Nie, M. (2022). Microbial respiratory thermal adaptation is regulated by r-/K-strategy dominance. Ecol Lett, 25(11), 2489-2499.

Daims, H., Lebedeva, E. V., Pjevac, P., Han, P., Herbold, C., Albertsen, M., Jehmlich, N., Palatinszky, M., Vierheilig, J., Bulaev, A. (2015) Complete nitrification by Nitrospira bacteria. Nature, 528(7583), 504-509.

Fierer, N., Bradford, M. A., & Jackson, R. B. (2007). Toward an ecological classification of soil bacteria. Ecology, 88(6), 1354-1364.

Fierer, N., Lauber, C. L., Ramirez, K. S., Zaneveld, J., Bradford, M. A., & Knight, R. (2012). Comparative metagenomic, phylogenetic and physiological analyses of soil microbial communities across nitrogen gradients. Isme j, 6(5), 1007-1017.

Francioli, D., Schulz, E., Lentendu, G., Wubet, T., Buscot, F., & Reitz, T. (2016). Mineral vs. Organic Amendments: Microbial Community Structure, Activity and Abundance of Agriculturally Relevant Microbes Are Driven by Long-Term Fertilization Strategies. Front Microbiol, 7, 1446.

Li, H., Yang, S., Semenov, M. V., Yao, F., Ye, J., Bu, R., Ma, R., Lin, J., Kurganova, I., Wang, X., Deng, Y., Kravchenko, I., Jiang, Y., Kuzyakov, Y. (2021). Temperature sensitivity of SOM decomposition is linked with a K-selected microbial community. Glob Chang Biol, 27(12), 2763-2779.

Li, H., Ye, D. D., Wang, X. G., Settles, M. L., Wang, J., Hao, Z. Q., Dong P., Jiang Y., Ma, Z. S. (2014). Soil bacterial communities of different natural forest types in Northeast China. Plant Soil, 383(1-2), 203-216.

Li, L. Y., Huang, D. Y., Hu, Y. X., Rudling, M. N., Canniffe, P. D., Wang F. P., Wang, Y. Z. (2023). Globally distributed Myxococcota with photosynthesis gene clusters illuminate the origin and evolution of a potentially chimeric lifestyle. Nat Commun, 14, 6450.

Männistö, M., Ganzert, L., Tiirola, M., Häggblom, M. M., & Stark, S. (2016). Do shifts in life strategies explain microbial community responses to increasing nitrogen in tundra soil? Soil Biol Biochem, 96, 216-228.

Stone, B. W. G., Dijkstra, P., Finley, B. K., Fitzpatrick, R., Foley, M. M., Hayer, M., Hofmockel, K. S., Koch, B. J., Li, J., Liu, X. J. A., Martinez, A., Mau, R. L., Marks, J., Monsaint-Queeney, V., Morrissey, E. M., Propster, J., Pett-Ridge, J., Purcell, A. M., Schwartz, E., Hungate, B. A. (2023). Life history strategies among soil bacteria-dichotomy for few, continuum for many. Isme j, 17(4), 611-619.

Van Kessel, M. A, H. J., Speth, D. R., Albertsen. M., Nielsen, P. H., Op den Camp, H. J. M., Kartal, B., Jetten, M. S. M., Lücker, S. (2015). Complete nitrification by a single microorganism. Nature, 528(7583), 555-559.

Zhou, Z. H., Wang, C. K., Jiang, L. F., & Luo, Y. Q. (2017). Trends in soil microbial communities during secondary succession. Soil Biol Biochem, 115, 92-99.
